# Supplementary material for: Functional 3D architecture in an intrinsically disordered E3 ligase domain facilitates ubiquitin transfer
Source: Nat Commun. 2020 Jul 30;11:3807. doi: 10.1038/s41467-020-17647-x (PMC7393505; doi:10.1038/s41467-020-17647-x)
Supplement: Supplementary file 1 — Supplementary Information [file 41467_2020_17647_MOESM1_ESM.pdf]

Supplementary information

Functional 3D architecture in an intrinsically disordered E3 ligase domain facilitates ubiquitin transfer

Paul Murphy[1], Yingqi Xu[2], Sarah L. Rouse[2], Ellis G. Jaffray[1], Anna Plechanovová[1], Steve J. Matthews[2], J Carlos Penedo[3, 4] and Ronald T. Hay[1]

[1]Centre for Gene Regulation and Expression, School of Life Sciences, University of Dundee, DD1 5EH, UK
[2]Centre for Structural Biology, Department of Life Sciences, Imperial College London SW7 2AZ, UK
[3]Centre of Biophotonics, School of Physics and Astronomy, University of St. Andrews, KY16 9SS, UK,
[4]Biomedical Sciences Research Complex, School of Biology, University of St. Andrews, KY16 9ST, UK

Corresponding author email: r.t.hay@dundee.ac.uk

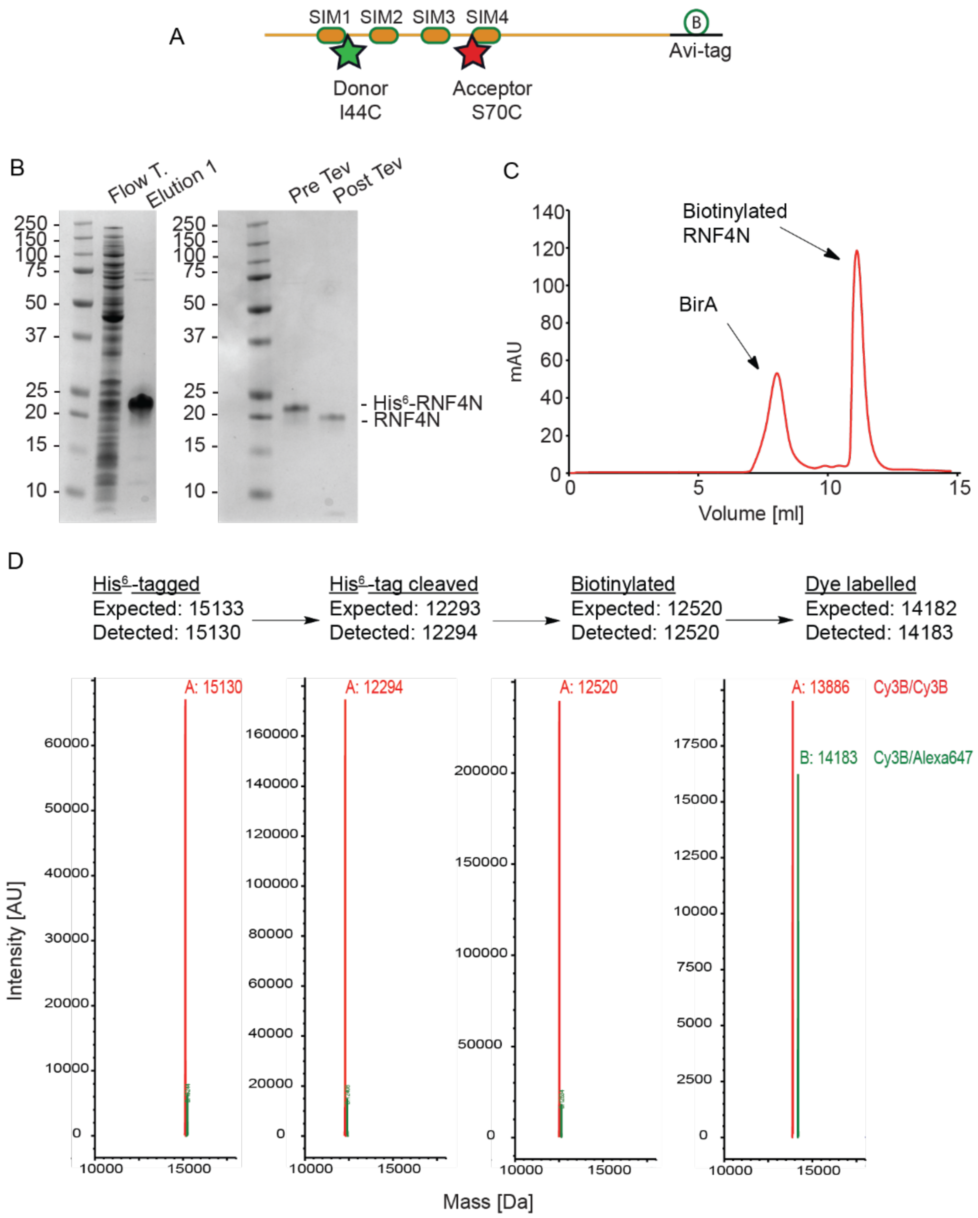

Supplementary figure 1. Production of RNF4N 44/70 peptide.

A, model of the N-terminal region of RNF4 bearing a C-terminal AviTag (RNF4N). The AviTag allows for site directed biotinylation of and internal lysine residue. The four SIMs are shown, along with the attachment position of the FRET fluorophore pair (residues 40, 70). B, RNF4N was initially expressed with an N-terminal His^6-tag to allow for purification. The His^6-tag was then cleaved off using TEV protease and assessed via SDS-PAGE. Purifications were carried out 3 times with similar results. C, the AviTag was then biotinylated and RNF4N-biotin purified via gel filtration. D, LCMS analysis was used at each stage of sample production (His^6-tag cleavage, biotinylation, FRET fluorophore conjugation). A number of variations of the RNF4N peptide were produced and validated following the same procedure.

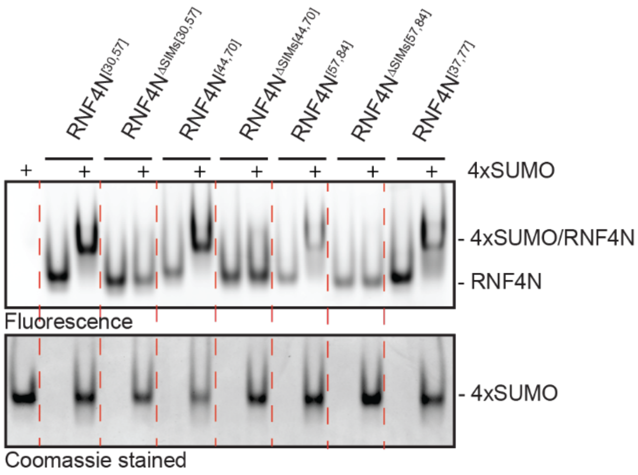

Supplementary figure 2. Binding between RNF4N SIM region peptides and 4xSUMO. Top panel, in-gel fluorescence analysis of the various RNF4N SIM region single-molecule peptides. RNF4N was incubated with/without 4xSUMO and then resolved via native PAGE electrophoresis. Samples incubated with 4xSUMO are highlighted above the gel image. Following in-gel fluorescence analysis the gels were then Coomassie stained to highlight the 4xSUMO, bottom panel.

Supplementary Table 1. Analysis of histograms figure 2B.

FRET efficiencies, relative populations and distribution-width values obtained with and without SUMO for RNF4N 30/57, RNF4N 44/70, RNF4N 57/84 and RNF4N 37/77 peptides. Values were calculated by non-linear squares fitting of the experimental single-molecule FRET histograms shown in figure 2B to one or two Gaussian distributions describing the number and relative contribution of FRET populations. The E_{FRET} magnitude was taken from the centre of the Gaussian and the error represents the standard error of the fit. The width of each Gaussian and its relative contribution are also shown. The value of ΔE_{FRET} was calculated only for the most populated FRET state as the difference between E_{FRET} in the presence of SUMO and E_{FRET} with no SUMO added.

SIM peptides	E_{FRET}	Width	%	ΔE_{FRET}	E_{FRET}	Width	%
**RNF4N 30/57**	0.642 ± 0.002	0.141 ± 0.002	95.2	-0.029 ± 0.003	0.28 ± 0.02	0.08± 0.02	4.8
**RNF4N 30/57 + SUMO**	0.613 ± 0.002	0.123 ± 0.032	100		--	--	--
**RNF4N 44/70**	0.693 ± 0.004	0.153 ± 0.001	75.5	-0.066 ± 0.005	0.34 ± 0.03	0.26± 0.07	24.5
**RNF4N 44/70 + SUMO**	0.627 ± 0.003	0.141 ± 0.006	100		--	--	--
**RNF4N 57/84**	0.727 ± 0.002	0.108 ± 0.004	81	-0.016 ± 0.003	0.35 ± 0.05	0.36 ± 0.15	19
**RNF4N 57/84 + SUMO**	0.711 ± 0.002	0.107 ± 0.004	100		--	--	--
**RNF4N 37/77**	0.394 ± 0.002	0.126 ± 0.005	100	0.004 ± 0.003	--	--	--
**RNF4N 37/77 + SUMO**	0.398 ± 0.002	0.146 ± 0.005	100		--	--	--

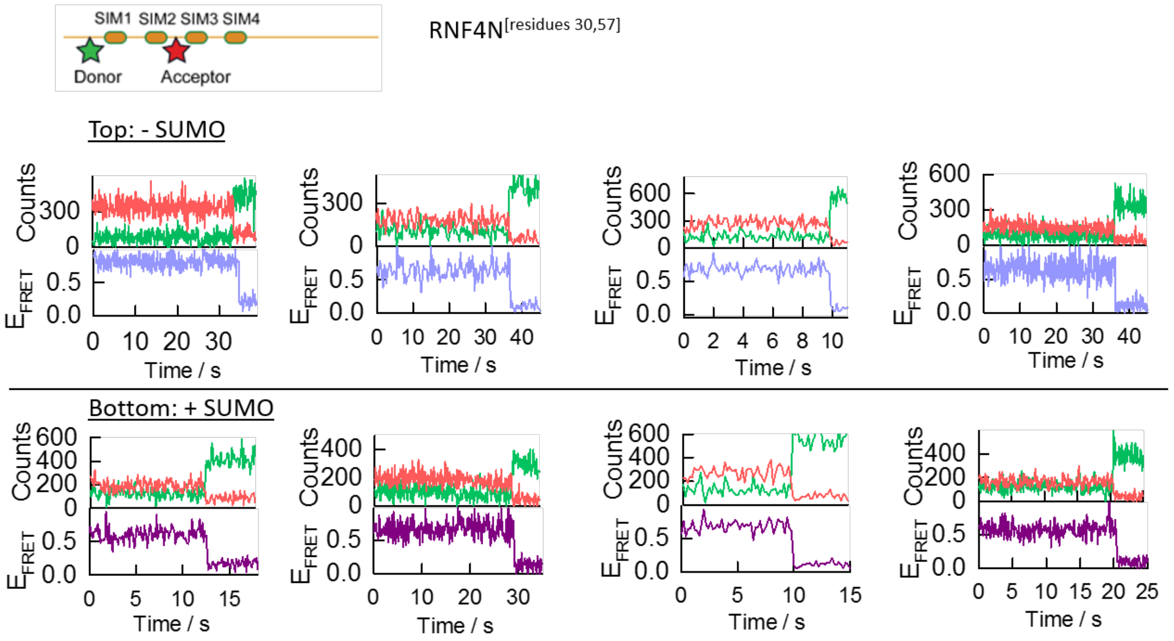

Supplementary figure 3. Representative single-molecule trajectories.

Representative single-molecule donor (green) and acceptor (red) intensity trajectories (top panels) and corresponding single-molecule FRET trace (bottom panels) obtained for the RNF4 peptide labelled at residues 30/57 in the presence (light blue) and absence (purple) of SUMO.

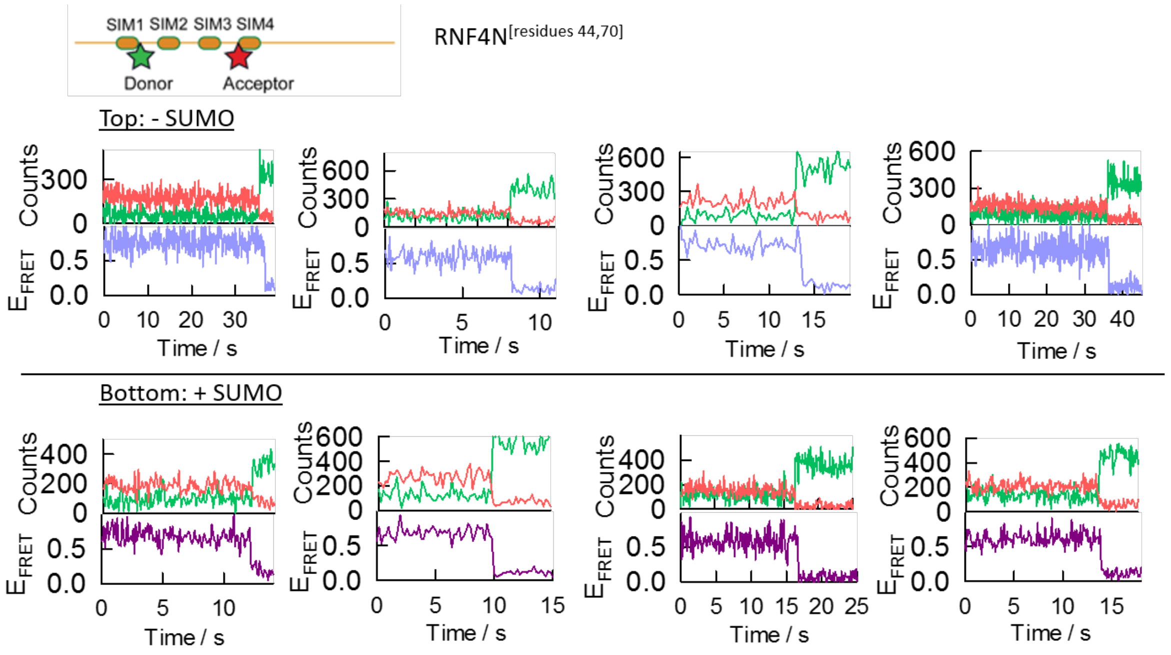

Supplementary figure 4. Representative single-molecule trajectories.

Representative single-molecule donor (green) and acceptor (red) intensity trajectories (top panels) and corresponding single-molecule FRET trace (bottom panels) obtained for the RNF4 peptide labelled at residues 44/70 in the presence (light blue) and absence (purple) of SUMO.

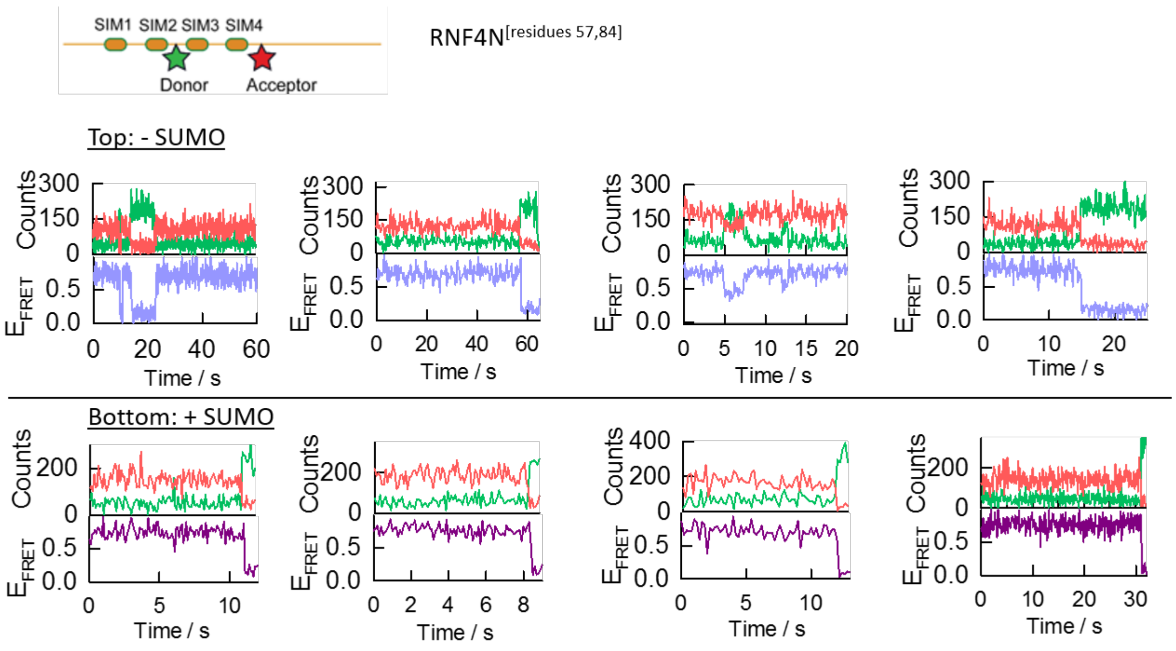

Supplementary figure 5. Representative single-molecule trajectories.

Representative single-molecule donor (green) and acceptor (red) intensity trajectories (top panels) and corresponding single-molecule FRET trace (bottom panels) obtained for the RNF4 peptide labelled at residues 57/84 in the presence (light blue) and absence (purple) of SUMO.

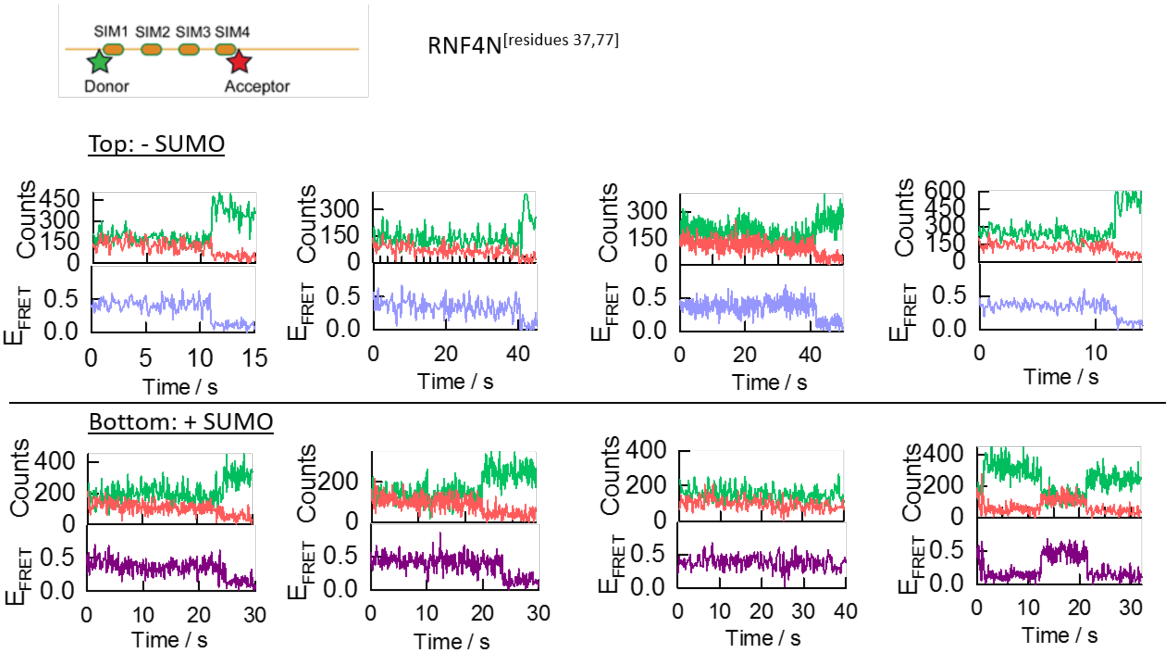

Supplementary figure 6. Representative single-molecule trajectories.

Representative single-molecule donor (green) and acceptor (red) intensity trajectories (top panels) and corresponding single-molecule FRET trace (bottom panels) obtained for the RNF4 peptide labelled at residues 37/77 in the presence (light blue) and absence (purple) of SUMO.

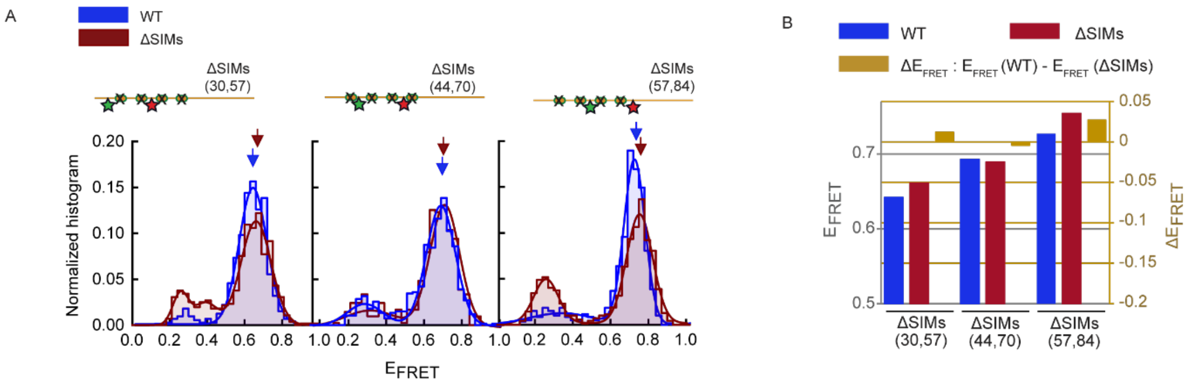

Supplementary figure 7. Role of SIMs in RNF4N conformation.

A, Single-molecule FRET histograms obtained from RNF4N WT (blue) and ΔSIMs mutant peptides (red) labelled with donor/acceptor FRET dyes (positions indicated inset). B, comparison of FRET efficiencies from the main FRET populations (arrows in A) for RNF4N$^{\Delta SIMs}$ (red), WT RNF4N (blue) and relative FRET efficiency changes (ΔE_{FRET}) between ΔSIM mutants and the corresponding WT (yellow).

Supplementary Table 2. Analysis of histograms from supplementary figure 7.

FRET efficiencies, relative populations and distribution-width values obtained for SIM mutant peptides RNF4N$^{\Delta SIMs}$ 30/57, RNF4N$^{\Delta SIMs}$ 44/70 and RNF4N$^{\Delta SIMs}$ 57/84. Values were calculated by non-linear squares fitting of the experimental single-molecule FRET histograms shown in supplementary figure 7 to one, two or three Gaussian distributions describing the number and relative contribution of FRET populations. The E_{FRET} magnitude was taken from the centre of the Gaussian and the error represents the standard error of the fit. The width of each Gaussian and its relative contribution are also shown.

Mutant peptides	E_{FRET}	Width	%	E_{FRET}	Width	%[2]	E_{FRET}	Width	%
**RNF4N$^{\Delta SIMs}$ 30/57**	0.661 ± 0.003	0.157 ± 0.008	75.4	0.26 ± 0.01	0.08 ± 0.02	12.1	0.39 ± 0.02	0.12 ± 0.05	12.5
**RNF4N$^{\Delta SIMs}$ 44/70**	0.689 ± 0.003	0.149 ± 0.007	81.7	0.29 ± 0.02	0.18 ± 0.05	18.3	--	--	--
**RNF4N$^{\Delta SIMs}$ 57/84**	0.754 ± 0.002	0.133 ± 0.005	72.3	0.259 ± 0.006	0.12 ± 0.01	27.7	--	--	--

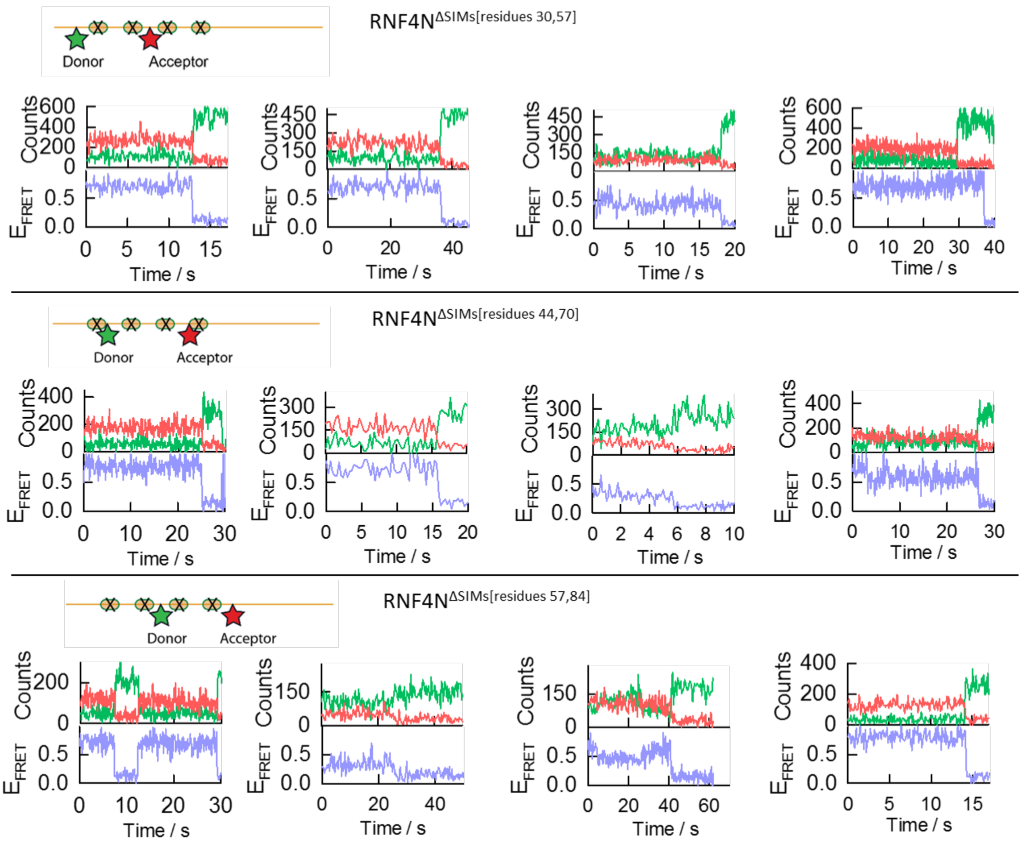

Supplementary figure 8. Representative single-molecule trajectories.

Representative single-molecule donor (green) and acceptor (red) intensity trajectories and corresponding single-molecule FRET trace (light blue) obtained for the RNF4N$^{\Delta SIMs}$ peptides (top, 30/57; middle, 44/70; bottom, 57/84).

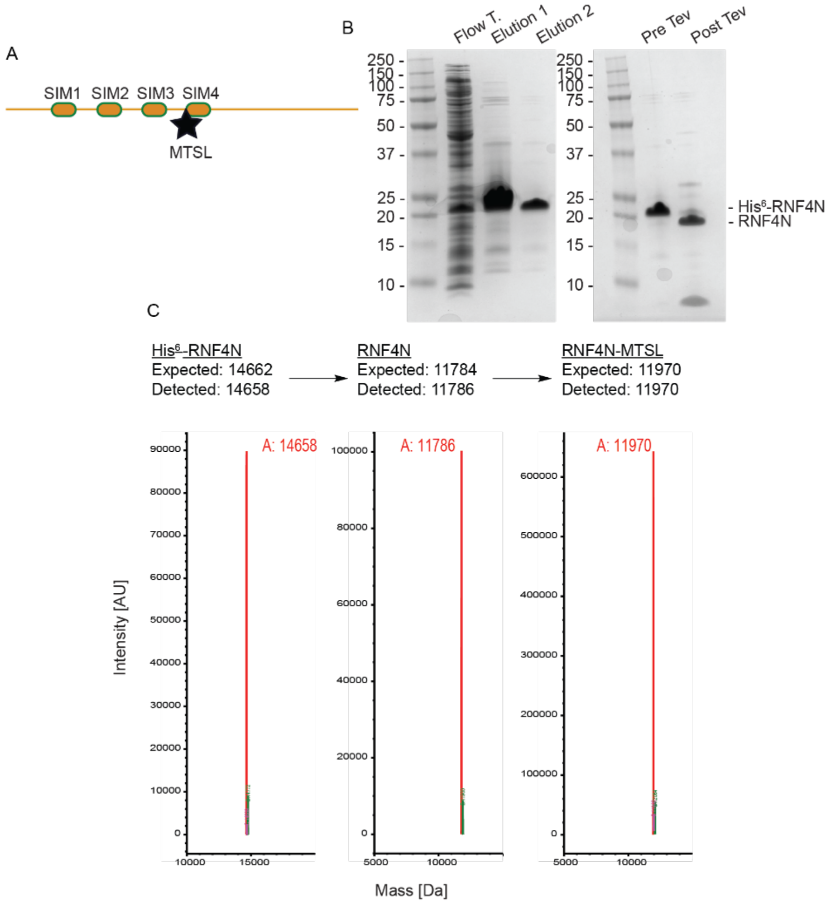

Supplementary figure 9. Production of ^{15}N-enriched RNF4N with MTSL spin label.

A, model of the N-terminal region of RNF4 (RNF4N) bearing a MTSL spin label next to SIM4 (residues 70). B, RNF4N was initially expressed with an N-terminal His^6-tag to allow for purification. The His^6-tag was was then cleaved off using TEV protease and assessed via SDS-PAGE. Purifications were carried out 3 times with similar results. C, LCMS analysis was used at each stage of sample production (His^6-tag cleavage, MTSL labelling). Each of the MTSL labelled ^{15}N-enriched RNF4N peptides were produced and validated following the same procedure.

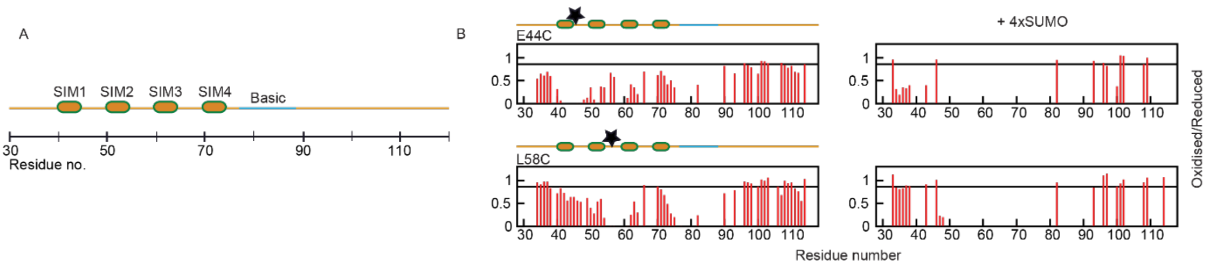

Supplementary figure 10. NMR PRE analysis of ^{15}N-enriched RNF4N with MTSL spin label.

A, RNF4N peptide layout used in B. B, histograms of HSQC peak intensity ratios plotted for RNF4N labelled at residues 44 or 58 (left) and in complex with SUMO chains (right). Peaks are absent due to severe peak broadening in the SUMO complex. Significant intensity reductions are below 0.85 (black line). Corresponding ^{1}H-^{15}N HSQC spectra are found in Supplementary figures 14 and 15.

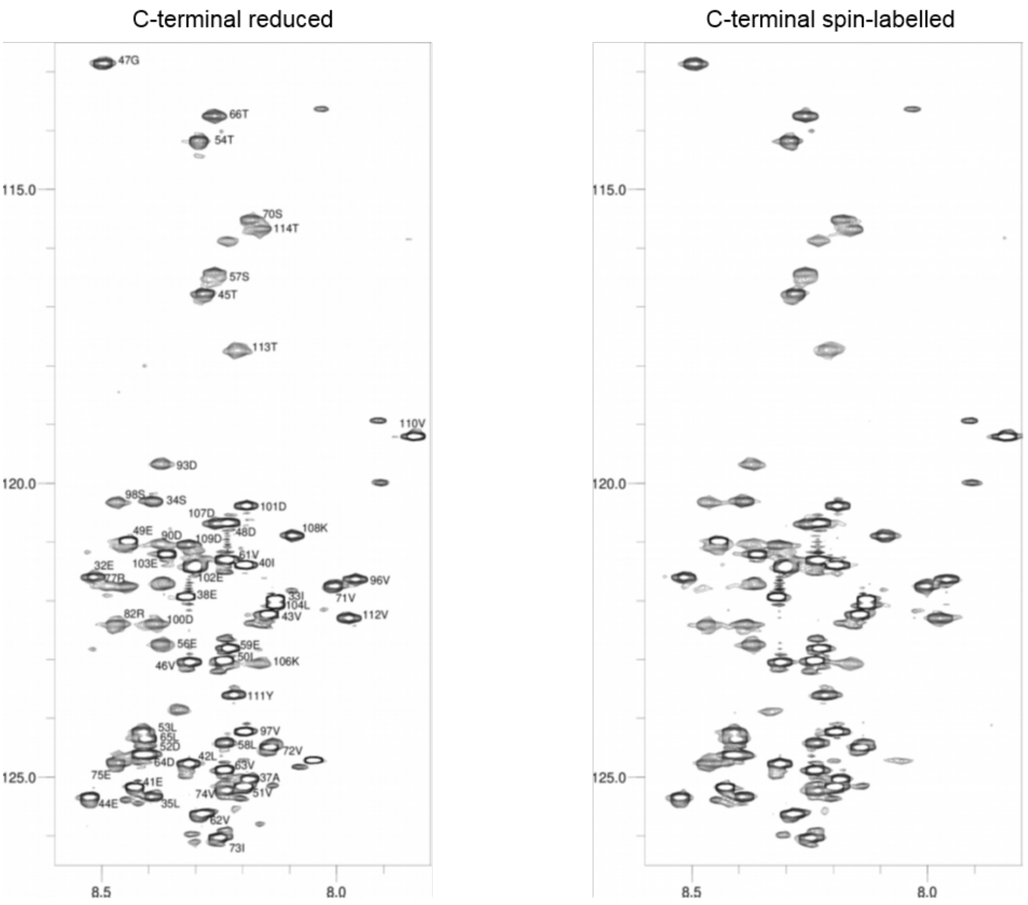

Supplementary figure 11. Spectra for C-terminal spin-labelled PRE peptides.

^{1}H-^{15}N HSQC NMR spectra for C-terminally spin-labelled RNF4N peptides. Spectra were recorded at 800 MHz spectrometer frequency. Relative intensities were measured for reduced

(left) and oxidised/paramagnetic (right) conditions to enable calculation of the paramagnetic relaxation enhancements (PREs).

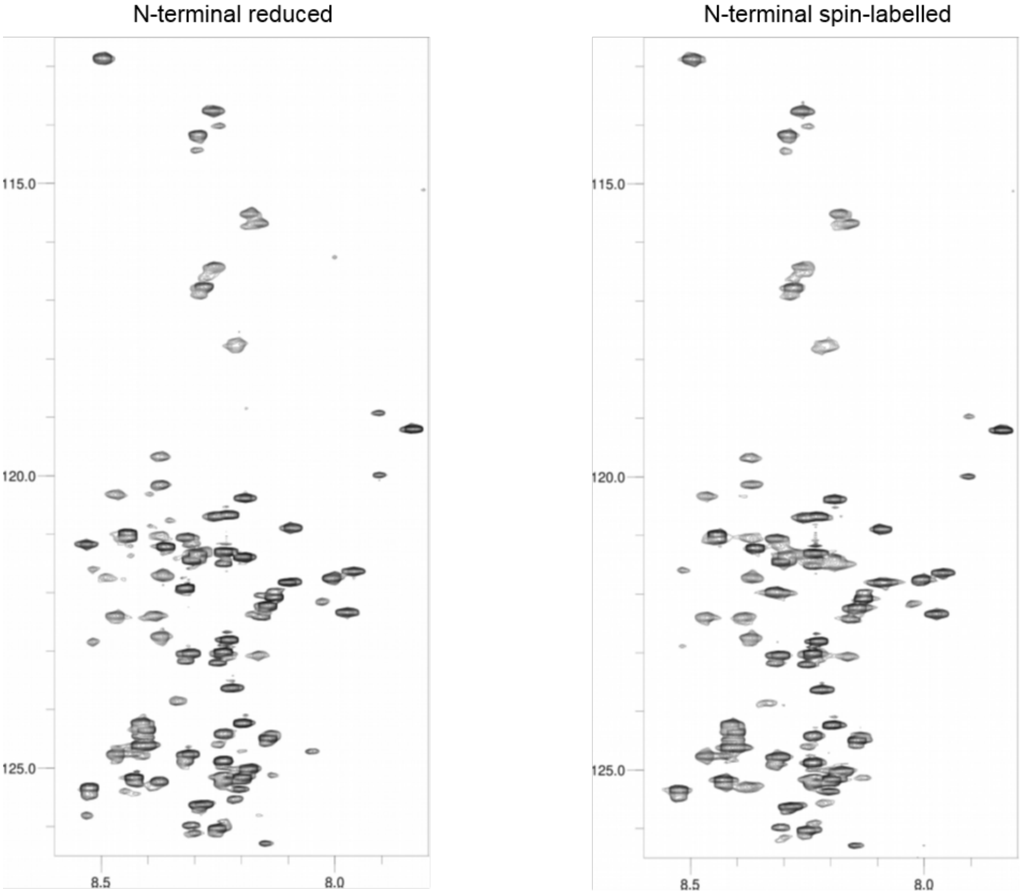

Supplementary figure 12. Spectra for N-terminal spin-labelled PRE peptides.

^{1}H-^{15}N HSQC NMR spectra for N-terminally spin-labelled RNF4N peptides. Spectra were recorded at 800 MHz spectrometer frequency. Relative intensities were measured for reduced (left) and oxidised/paramagnetic (right) conditions to enable calculation of the paramagnetic relaxation enhancements (PREs).

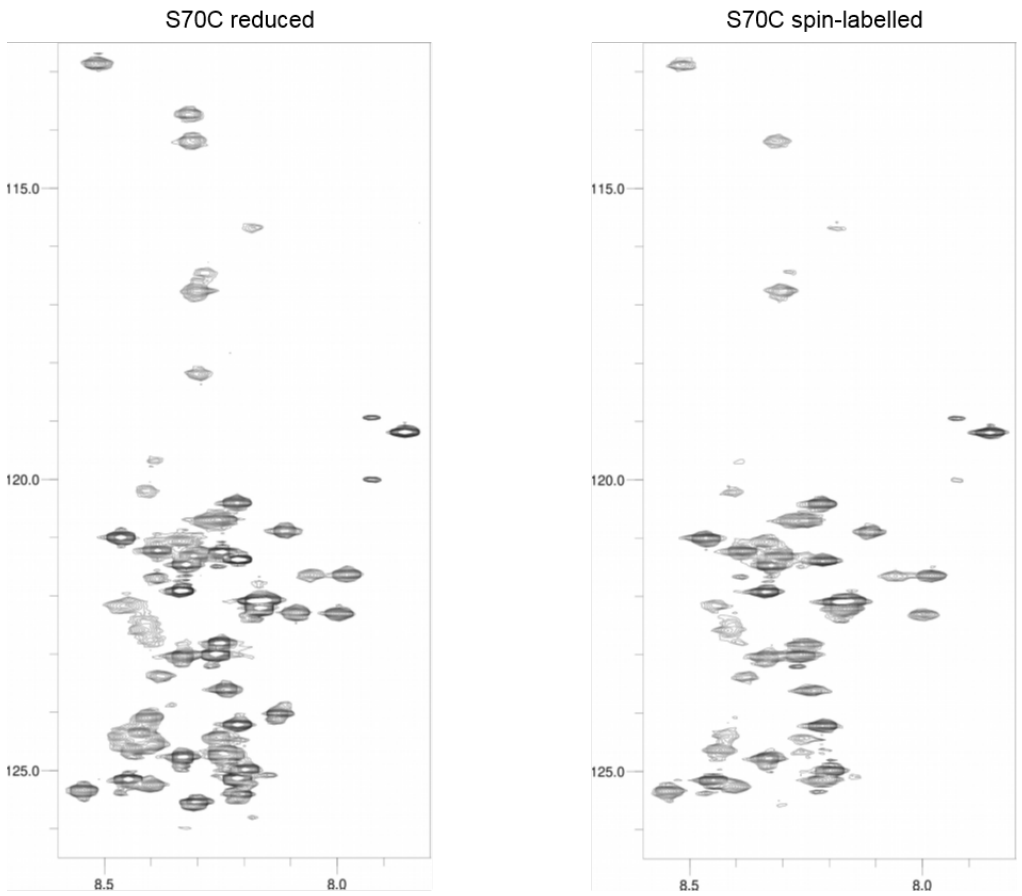

Supplementary figure 13. Spectra for S70C spin-labelled PRE peptides.

^{1}H-^{15}N HSQC NMR spectra for S70C spin-labelled RNF4N peptides. Spectra were recorded at 600 MHz spectrometer frequency. Relative intensities were measured for reduced (left) and oxidised/paramagnetic (right) conditions to enable calculation of the paramagnetic relaxation enhancements (PREs).

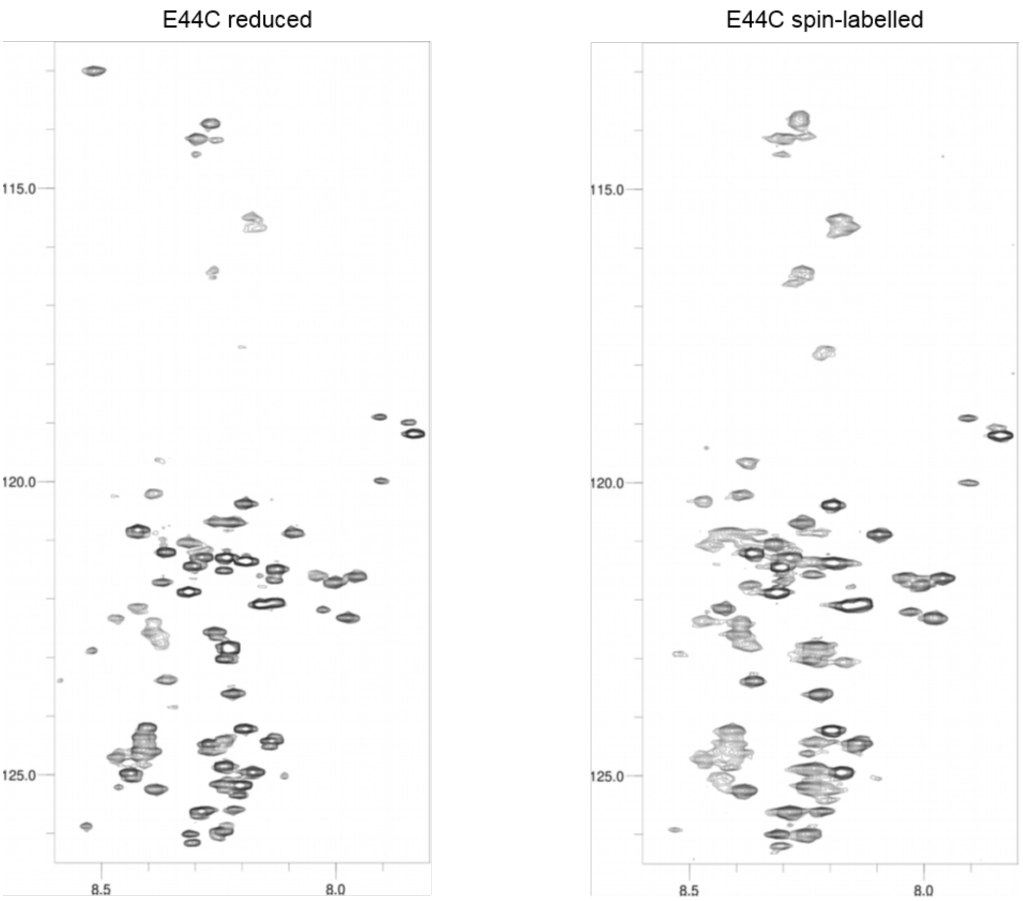

Supplementary figure 14. Spectra for E44C spin-labelled PRE peptides.

^{1}H-^{15}N HSQC NMR spectra for E44C spin-labelled RNF4N peptides. Spectra were recorded at 800 MHz spectrometer frequency. Relative intensities were measured for reduced (left) and oxidised/paramagnetic (right) conditions to enable calculation of the paramagnetic relaxation enhancements (PREs).

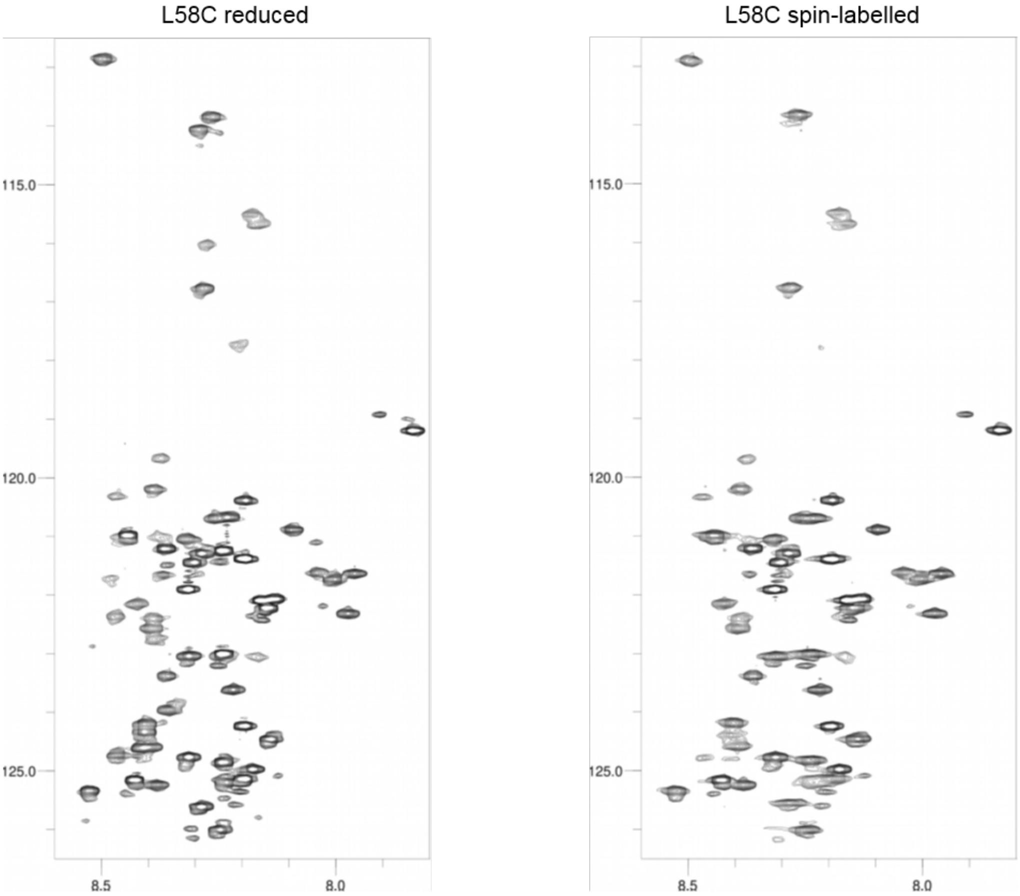

Supplementary figure 15. Spectra for L58C spin-labelled PRE peptides.

^{1}H-^{15}N HSQC NMR spectra for L58C spin-labelled RNF4N peptides. Spectra were recorded at 800 MHz spectrometer frequency. Relative intensities were measured for reduced (left) and oxidised/paramagnetic (right) conditions to enable calculation of the paramagnetic relaxation enhancements (PREs).

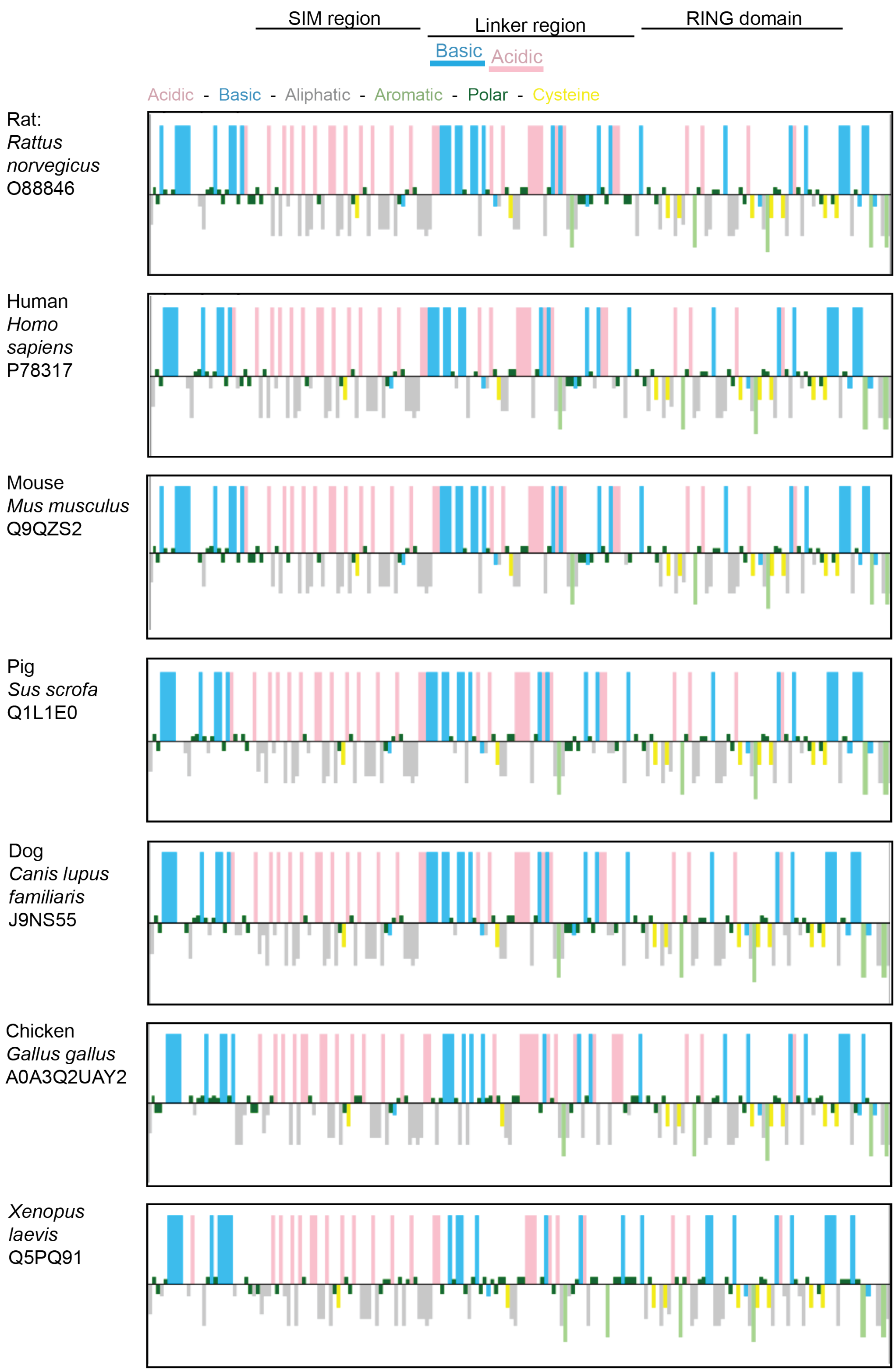

Supplementary figure 16. Conservation of charge segregation in RNF4 across species. Hydropathy plots displaying charge characteristics of amino acid side chains for RNF4, with each species displayed inset. The hydrophobic SIMs are embedded in a highly acidic background. The SIMs are then followed by a basic arginine-rich motif, which is in turn followed by a stretch of acidic residues.

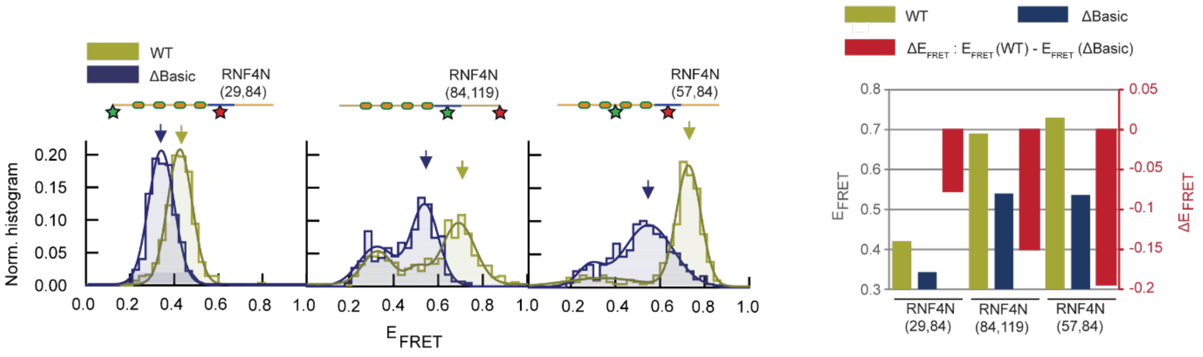

Supplementary figure 17. Role of basic region in RNF4N compaction.

Left, single-molecule FRET histograms from RNF4N WT (yellow) and ΔBasic peptides (blue) labelled with donor/acceptor FRET dyes (position indicated inset). Right, relative change (red) in FRET efficiency (ΔE_{FRET}) between each ΔBasic mutant (blue) and corresponding WT peptide (yellow), as highlighted by the arrows in histograms. Single-molecule FRET histograms are normalised to area unity and built from 400-700 molecules.

Supplementary Table 3. Analysis of histograms from supplementary figure 17.

FRET efficiencies, relative populations and distribution-width values obtained for basic region mutant peptides RNF4$^{\Delta Basic}$ 29/84, RNF4$^{\Delta Basic}$ 84/119 and RNF4N$^{\Delta Basic}$ 57/84. Values were calculated by non-linear squares fitting of the experimental single-molecule FRET histograms shown in supplementary figures 17 to one, two or three Gaussian distributions describing the number and relative contribution of FRET populations. The E_{FRET} magnitude was taken from the centre of the Gaussian and the error represents the standard error of the fit. The width of each Gaussian and its relative contribution are also shown. The value of ΔE_{FRET} was calculated as the difference between the E_{FRET} for the most populated FRET state at each experimental condition.

Mutant peptides	E_{FRET}	Width	%	ΔE_{FRET}	E_{FRET}	Width	%	E_{FRET}	Width	%
**RNF4 29/84**	0.427 ± 0.001	0.109 ± 0.002	100	-0.084 ± 0.002	--	--	--	--	--	--
**RNF4$^{\Delta Basic}$ 29/84**	0.343 ± 0.002	0.115 ± 0.003	100							
**RNF4 84/119**	0.691 ± 0.001	0.15 ± 0.01	60.9	-0.15 ± 0.006	0.33 ± 0.01	0.13 ± 0.03	29.4	0.50 ± 0.02	0.09 ± 0.04	9.6
**RNF4$^{\Delta Basic}$ 84/119**	0.32 ± 0.01	0.16 ± 0.03	38.9					0.540 ± 0.006	0.12 ± 0.01	61.1
**RNF4N 57/84**	0.727 ± 0.002	0.108 ± 0.004	81	-0.19 ± 0.006	0.35 ± 0.05	0.36 ± 0.15	19			
**RNF4$^{\Delta Basic}$ 57/84**					0.25 ± 0.01	0.07 ± 0.02	7	0.537 ± 0.006	0.24 ± 0.01	93

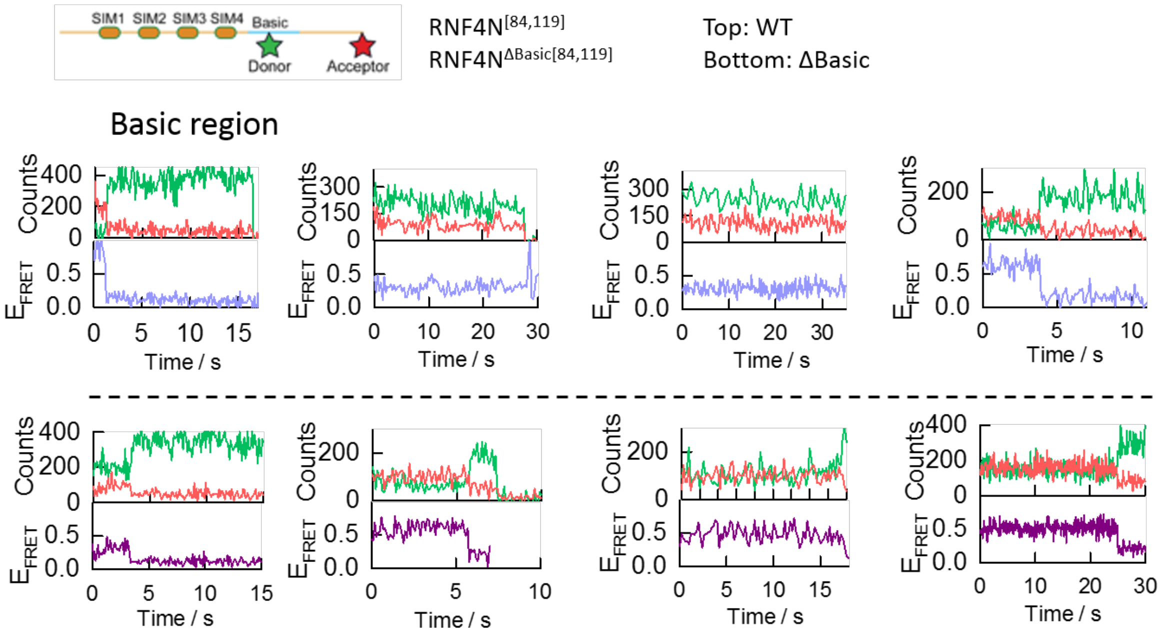

Supplementary figure 18. Representative single-molecule trajectories.

Representative single-molecule donor (green) and acceptor (red) intensity trajectories (top panels) and corresponding single-molecule FRET trace (bottom panels) obtained for RNF4N 84/119 peptide (light blue; top traces) and ΔBasic mutated version (purple; bottom traces).

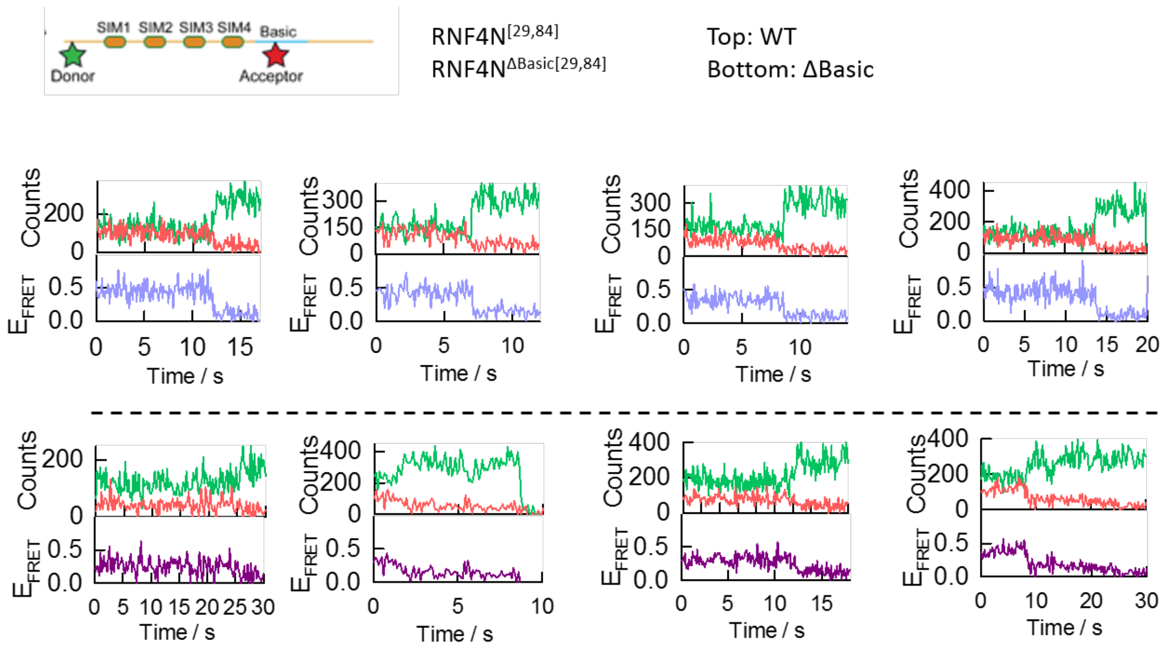

Supplementary figure 19. Representative single-molecule trajectories.

Representative single-molecule donor (green) and acceptor (red) intensity trajectories (top panels) and corresponding single-molecule FRET trace (bottom panels) obtained for RNF4N 29/84 peptide (light blue; top traces) and ΔBasic mutated version (purple; bottom traces).

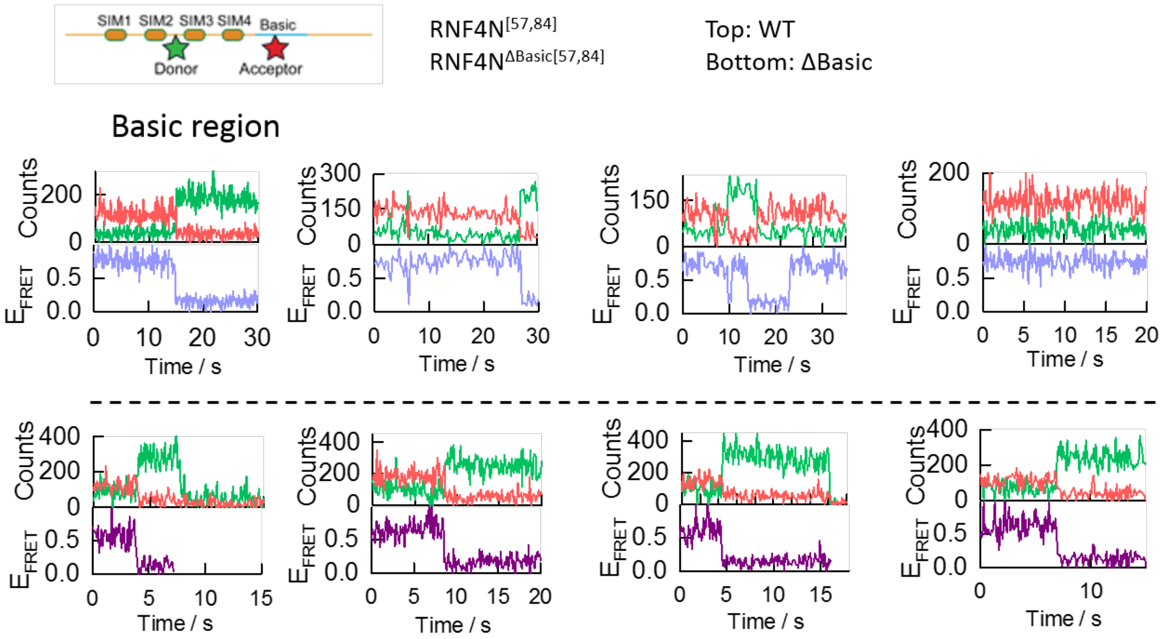

Supplementary figure 20. Representative single-molecule trajectories.

Representative single-molecule donor (green) and acceptor (red) intensity trajectories (top panels) and corresponding single-molecule FRET trace (bottom panels) obtained for RNF4N 57/84 peptide (light blue; top traces) and ΔBasic mutated version (purple; bottom traces).

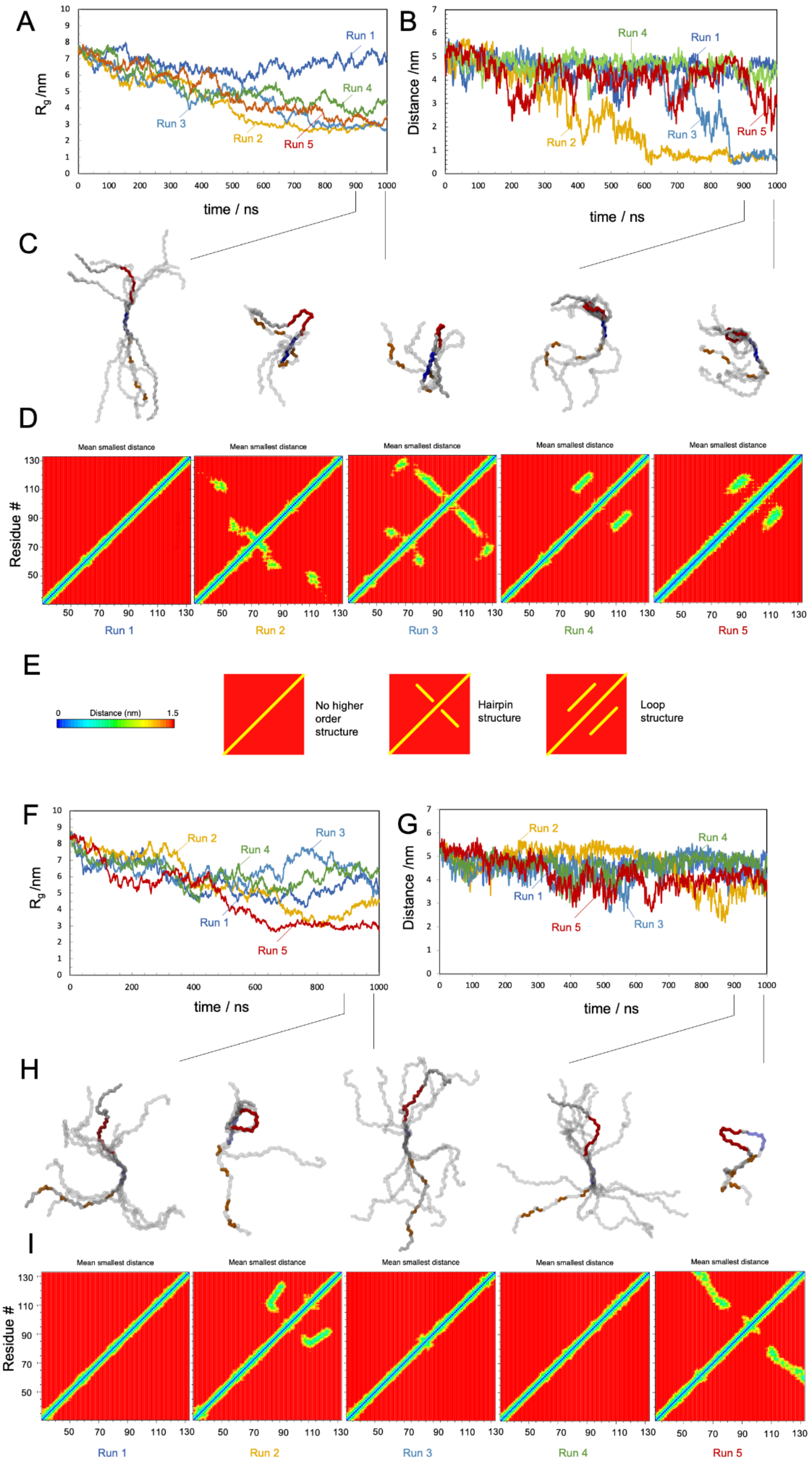
A
B
C
D
E
F
G
H
I
R_g /nm
Distance /nm
time / ns
Run 1
Run 2
Run 3
Run 4
Run 5
Mean smallest distance
Residue #
Distance (nm)
No higher order structure
Hairpin structure
Loop structure

Supplementary figure 21. Comparison of properties derived from molecular simulations of RNF4N WT and ΔBasic peptides.

A, radius of gyration over time for each of 5 independent simulations of RNF4N WT. B, distance between SIM2/3 (defined by residue E59) and the midpoint of the arginine-rich motif (residues 77-88) over time, of RNF4N WT. C, conformational clustering of RNF4N WT over the final 100 ns of each trajectory. D, residue-residue contact plots of RNF4N WT. E, schematic showing contact patterns and how they correspond to structure defined as loops or hairpins. F, radius of gyration over time for each of 5 independent simulations of RNF4N ΔBasic. G, distance between SIM2/3 (defined by residue E59) and the midpoint of the arginine-rich motif over time, of RNF4N ΔBasic. H, conformational clustering of RNF4N ΔBasic over the final 100 ns of each trajectory. I, residue-residue contact plots of RNF4N ΔBasic.

Supplementary table 4. Properties of RNF4N simulations for wildtype and ΔBasic RNF4N. Radius gyration calculated for all backbone beads. Distances correspond to the N-terminus residue 33, the midpoint residue 80, and the C-terminus residue 132. Standard deviations shown in parentheses. Table corresponds to Supplementary figure 18.

	**Run number**	**Radius gyration**	**% Increase buried surface area**	**Distance Nter-to-Cter**	**Distance Nter-to-mid**	**Distance Mid-to-Cter**
**Wildtype**	1	6.6 (0.5)	3.1 (0.6)	7.2 (2.6)	9.6 (1.2)	10.7 (1.7)
	2	2.9 (0.3)	9.5 (1.1)	4.6 (2.1)	6.1 (1.6)	5.4 (1.5)
	3	3.6 (0.8)	8.6 (4.3)	9.6 (1.3)	7.8 (1.5)	4.2 (0.9)
	4	4.5 (0.5)	5.1 (5.4)	12.9 (2.4)	8.5 (1.9)	6.5 (0.8)
	5	3.7 (0.4)	6.1 (5.0)	8.1 (1.7)	6.3 (1.5)	7.6 (0.9)
	mean	4.1 (1.4)	6.3 (2.8)	8.5 (3.4)	7.7 (2.0)	6.9 (2.5)
**ΔBasic**	1	5.1 (0.5)	3.8 (1.2)	9.1 (1.8)	9.5 (1.6)	8.6 (2.2)
	2	4.3 (0.8)	12.8 (2.3)	9.4 (1.9)	7.7 (2.4)	6.2 (0.4)
	3	6.4 (0.7)	3.0 (1.0)	14.9 (2.3)	9.8 (1.6)	10.2 (2.0)
	4	5.9 (0.5)	2.9 (1.0)	9.5 (3.2)	10.0 (1.2)	10.1 (1.6)
	5	3.2 (0.4)	12.5 (7.8)	7.3 (1.8)	7.7 (1.1)	6.0 (1.2)
	mean	5.0 (1.3)	7.0 (3.9)	10.1 (3.4)	8.9 (1.9)	8.3 (2.4)

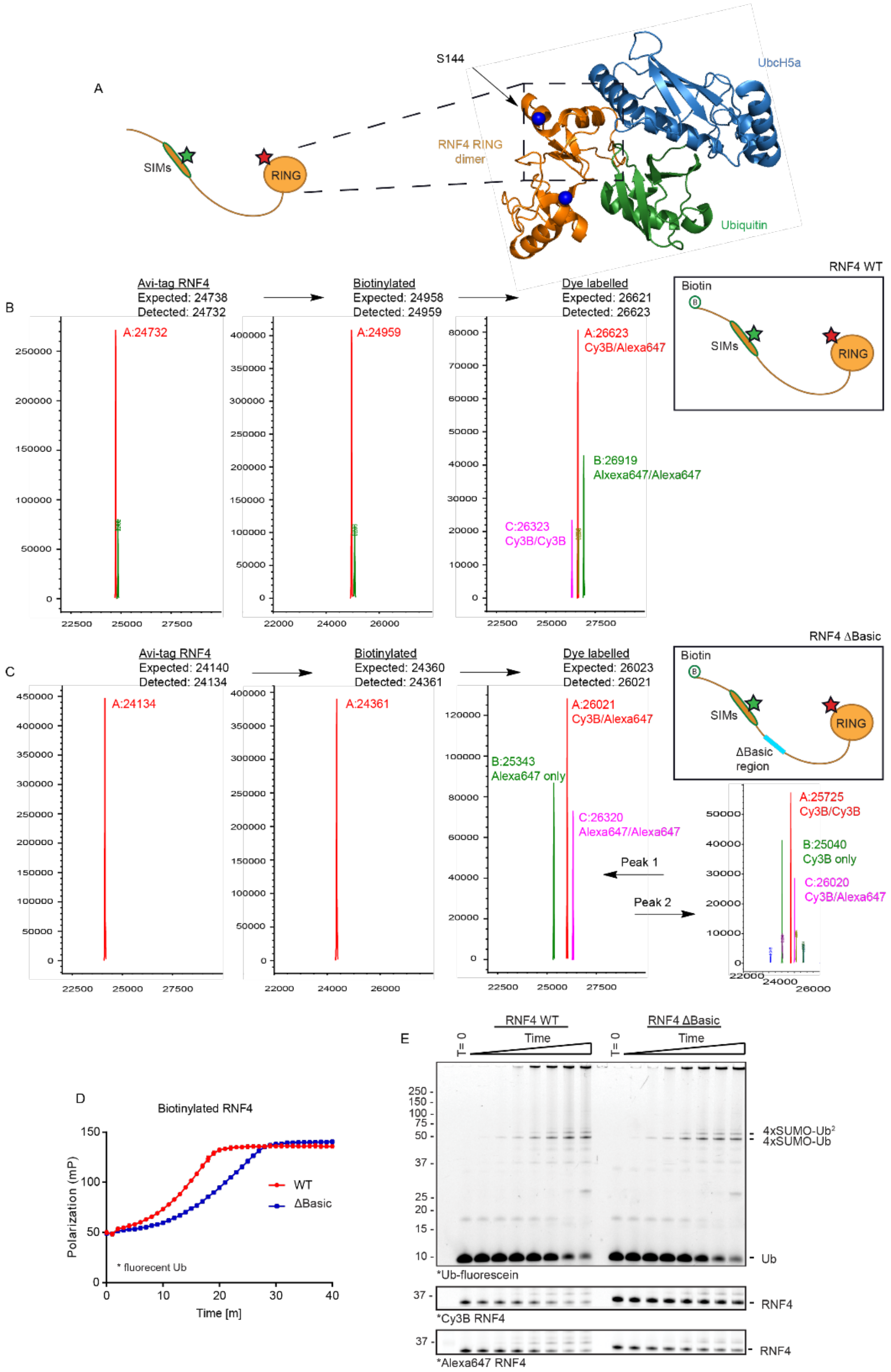

Supplementary figure 22. Production of full length RNF4 for smFRET analysis.

A left, illustration of RNF4 showing the position of the FRET dyes. A right, section of structure PDB: 4AP4 showing RNF4 RING dimer (orange) bound to an E2~Ub conjugate (blue and green respectively). The serine 144 residue in the RING domain that was mutated to cysteine for maleimide dye labelling is highlighted as a dark blue sphere. B, LCMS analysis of production of full length RNF4 WT for single-molecule FRET analysis. Each stage of production is validated including biotinylation and dye labelling. Due to stochastic dye

labelling three species are observed, donor/donor, donor/acceptor and acceptor/acceptor (donor, Cy3B; acceptor, Alexa647). The donor/acceptor labelled molecules are then selected for during analysis. C, same as B but production of ΔBasic version of full length RNF4. LCMS shows some under-labelling, with only donor or acceptor. D, fluorescence polarization real-time ubiquitination assay comparing activity of WT (red) against ΔBasic (blue) biotinylated RNF4. Assays contained 4xSUMO, E1, UbcH5a and fluorescent ubiquitin. Reactions initiated with ATP addition and FP measured over 40 minutes (experiments were carried out in triplicate, n=3). E, ubiquitination activity of FRET dye labelled RNF4 WT and ΔBasic mutant in response to increasing substrate (4xSUMO) concentrations. Assays (15 min) contained E1 activating enzyme, UbcH5a and fluorescently labelled ubiquitin. T = 0 is without ATP. Reaction products were fractionated by SDS-PAGE, followed by in-gel fluorescence. Main panel shows fluorescent ubiquitin. Bottom panels show RNF4 fluorescence corresponding to the indicated fluorescent dye. The experiments in D and E were carried out 3 times with similar results.

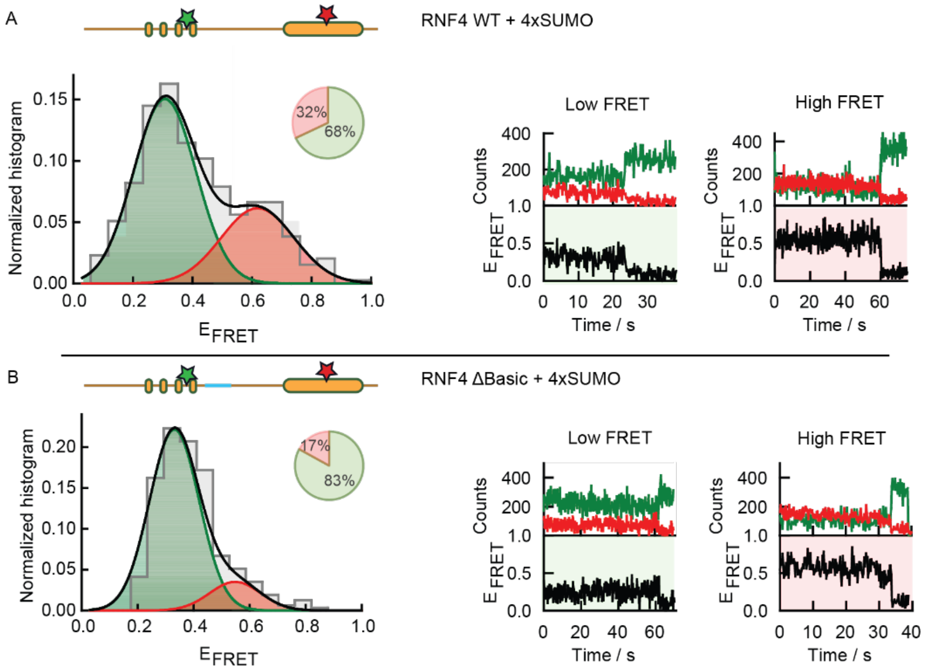

Supplementary figure 23. smFRET analysis of RNF4 with addition of SUMO chains.
A left, single-molecule FRET histograms obtained from full length RNF4 WT, labelled with donor/acceptor FRET dyes at SIMs and RING domain (illustrated inset). Pie charts indicate the percentage of molecules in a low FRET (green) or high FRET (red) state. A right, representative single-molecule trajectories obtained for RNF4 WT. **B**, as in A but analysis

performed on RNF4 ΔBasic. Single-molecule histograms were built from more than 300 molecules.

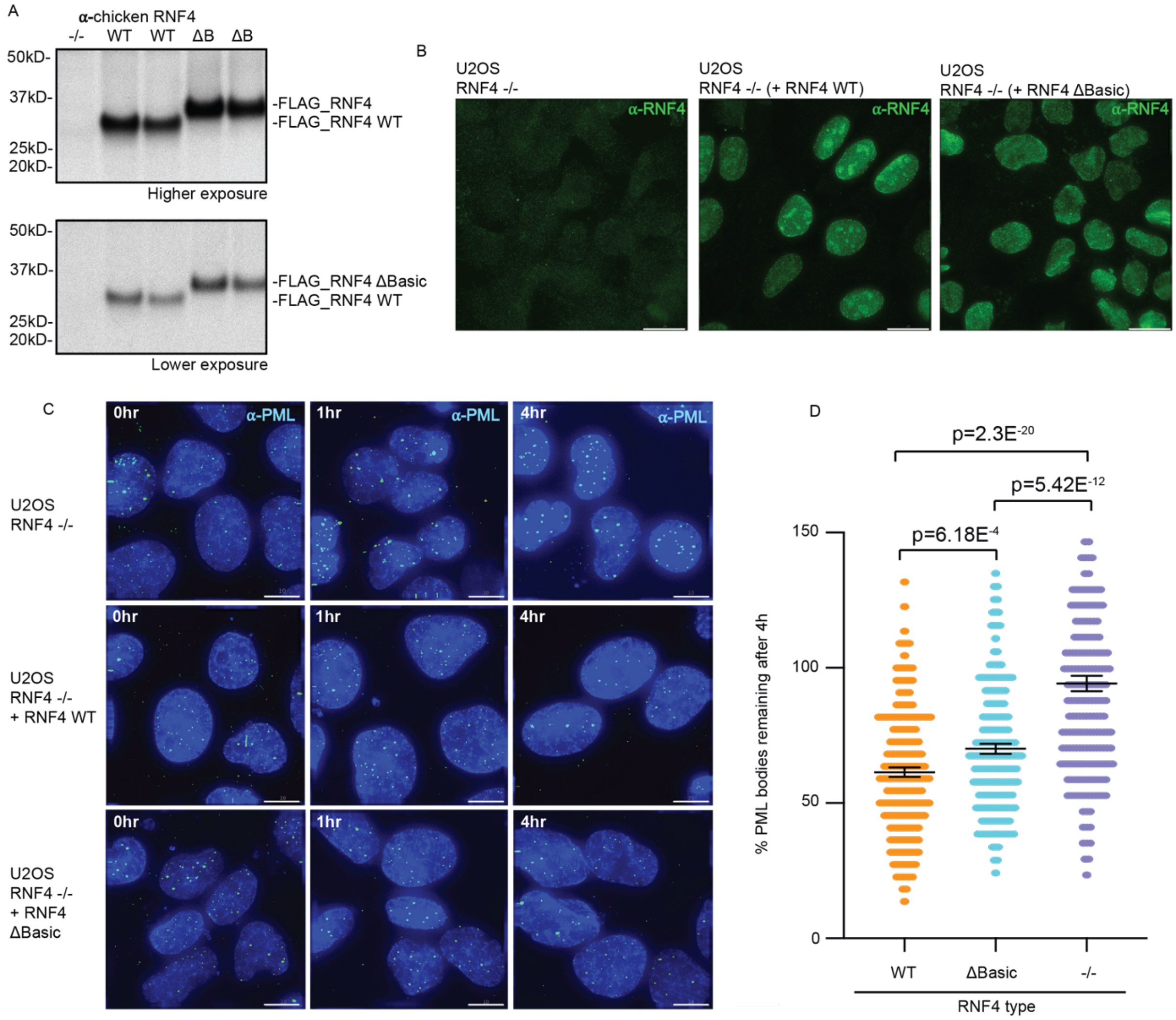

Supplementary figure 24. Delay in arsenic induced PML clearance by RNF4 ΔBasic.
A, U2OS RNF4 -/- cell line was stably transfected with FLAG-tagged RNF4 WT or RNF4 ΔBasic. RNF4 protein levels were analysed by western blotting using antibodies against RNF4. These experiments were carried out 3 times with similar results. B, U2OS cell lines were immunostained to assess expression of RNF4 (left, -/-; middle, RNF4 WT; right, RNF4 ΔBasic). Scale bars represent 20 μm. These experiments were carried out 3 times with similar results. C, U2OS cell lines were treated with arsenic and fixed after 1 and 4 hours. Cells were immunostained against PML. Scale bars represent 10 μm. D, Scatter plot showing the % PML bodies remaining at 4 hours after arsenic treatment in U2OS RNF4 -/- cell lines, transfected with either RNF4 wild-type or RNF4 ΔBasic (WT n=196, ΔBasic n=200, no plasmid n=200, where n = number of cells counted). Data were calculated by expressing counts of PML bodies at 4 hours as a % of the average counts at 1 hour. p-value calculated by two tailed unpaired t-

test assuming equal variances for WT versus ΔBasic (F-test p-value <0.421), and unpaired t-test with Welch's correction for any comparison with -/- (F-test p-value <0.0001). Data are presented as mean values +/- one SEM: WT = 61.4%, ΔBasic = 70.1% and -/- = 94.2%. 16 data points representing outliers with high % values were omitted from the plot.
