# Supplementary material for: Functional 3D architecture in an intrinsically disordered E3 ligase domain facilitates ubiquitin transfer
Source: Nat Commun. 2020 Jul 30;11:3807. doi: 10.1038/s41467-020-17647-x (PMC7393505; doi:10.1038/s41467-020-17647-x)
Supplement: Supplementary file 3 — Reporting Summary [file 41467_2020_17647_MOESM3_ESM.pdf]

nature research

Corresponding author(s): R. T. Hay

Last updated by author(s): Jul 3, 2020

Reporting Summary

Nature Research wishes to improve the reproducibility of the work that we publish. This form provides structure for consistency and transparency in reporting. For further information on Nature Research policies, see our Editorial Policies and the Editorial Policy Checklist.

Statistics

For all statistical analyses, confirm that the following items are present in the figure legend, table legend, main text, or Methods section.

n/a	Confirmed	
☐	☒	The exact sample size (n) for each experimental group/condition, given as a discrete number and unit of measurement
☐	☒	A statement on whether measurements were taken from distinct samples or whether the same sample was measured repeatedly
☐	☒	The statistical test(s) used AND whether they are one- or two-sided<br>*Only common tests should be described solely by name; describe more complex techniques in the Methods section.*
☒	☐	A description of all covariates tested
☒	☐	A description of any assumptions or corrections, such as tests of normality and adjustment for multiple comparisons
☐	☒	A full description of the statistical parameters including central tendency (e.g. means) or other basic estimates (e.g. regression coefficient) AND variation (e.g. standard deviation) or associated estimates of uncertainty (e.g. confidence intervals)
☐	☒	For null hypothesis testing, the test statistic (e.g. F, t, r) with confidence intervals, effect sizes, degrees of freedom and P value noted<br>*Give P values as exact values whenever suitable.*
☒	☐	For Bayesian analysis, information on the choice of priors and Markov chain Monte Carlo settings
☒	☐	For hierarchical and complex designs, identification of the appropriate level for tests and full reporting of outcomes
☒	☐	Estimates of effect sizes (e.g. Cohen's d, Pearson's r), indicating how they were calculated

Our web collection on statistics for biologists contains articles on many of the points above.

Software and code

Policy information about availability of computer code

Data collection	image processing: IDL: https://www.harrisgeospatial.com/Support/Self-Help-Tools/Help-Articles/Help-Articles-Detail/ArtMID/10220/ArticleID/15066/Install-and-License-IDL-86#
Data analysis	- modelling: FRET Positioning and Screening (FPS) software V1.1 : http://www.mpc.hhu.de/en/software/fps.html<br>- image analysis: tir2009: McCluskey, K., Shaw, E., Lafontaine, D. A., and Penedo, J. C. (2014) Single-molecule fluorescence of nucleic acids. Methods in molecular biology 1076, 759-791<br>- CCPN analysis 2.4: https://www.ccpn.ac.uk/v2-software/downloads<br>MD simulations:<br>- Modeller v9: Sali, A., and Blundell, T. L. (1993) Comparative protein modelling by satisfaction of spatial restraints.Journal of molecular biology 234, 779-815<br>- PDB2PQR server http://server.poissonboltzmann.org/pdb2pqr/ : Dolinsky, T. J., Czodrowski, P., Li, H., Nielsen, J. E., Jensen, J. H., Klebe, G., and Baker, N. A. (2007) PDB2PQR: expanding and upgrading automated preparation of biomolecular structures for molecular simulations. Nucleic acids research 35, W522-W525<br>- SIRAH 2.0: Machado, M. R., and Pantano, S. (2016) SIRAH tools: mapping, backmapping and visualization of coarse-grained models. Bioinformatics 32, 1568-1570<br>- GROMACS v5: http://www.gromacs.org<br>- VDM: Humphrey, W., Dalke, A., and Schulten, K. (1996) VMD: Visual molecular dynamics. Journal of Molecular Graphics 14, 33-38

For manuscripts utilizing custom algorithms or software that are central to the research but not yet described in published literature, software must be made available to editors and reviewers. We strongly encourage code deposition in a community repository (e.g. GitHub). See the Nature Research guidelines for submitting code & software for further information.

April 2020

Data

Policy information about availability of data

All manuscripts must include a data availability statement. This statement should provide the following information, where applicable:

- Accession codes, unique identifiers, or web links for publicly available datasets
- A list of figures that have associated raw data
- A description of any restrictions on data availability

Source data are provided with this paper and can be accessed at. https://doi.org/10.17630/5d766785-60c8-4dbe-99ec-926e2fa33798. Source data for figures 2-7 are available via this link.

Field-specific reporting

Please select the one below that is the best fit for your research. If you are not sure, read the appropriate sections before making your selection.

☒ Life sciences ☐ Behavioural & social sciences ☐ Ecological, evolutionary & environmental sciences

For a reference copy of the document with all sections, see nature.com/documents/nr-reporting-summary-flat.pdf

Life sciences study design

All studies must disclose on these points even when the disclosure is negative.

Sample size	The single-molecule FRET histograms were generated by adding molecules from consecutive movies of the same sample at different field of views within the microscope slides, and fitting the resulting histogram to one or two gaussian distributions, depending on the specific construct. The number of molecules included in the analysis was considered statistically adequate when the centre, width and relative contribution of each gaussian reached values that were unchanged by the addition of more molecules. In general, this condition was achieved when the single-molecule histograms contained 500 molecules or more.<br>All gel electrophoresis experiments were done in triplicate.
Data exclusions	During single molecule analysis background signal of about 0.1 FRET efficiency is subtracted as this represents the background.
Replication	All attempts at replication were successful. Experiments were carried out at least three times.
Randomization	In NMR and single molecule experiments samples were processed one sample at a time and therefore randomization was not done. Analysis by gel electrophoresis required that samples were loaded on to the gel in the correct order and thus it was inappropriate to randomize.
Blinding	Blinding was used in the immunofluorescence experiment reported in Supplementary figure 24. All samples were provided with a number and multiple images were collected from each cover slip. These images were saved and given a code and all counting of PML body numbers was carried out using the code.

Reporting for specific materials, systems and methods

We require information from authors about some types of materials, experimental systems and methods used in many studies. Here, indicate whether each material, system or method listed is relevant to your study. If you are not sure if a list item applies to your research, read the appropriate section before selecting a response.

Materials & experimental systems

n/a	Involved in the study	
☐	☒	Antibodies
☐	☒	Eukaryotic cell lines
☒	☐	Palaeontology and archaeology
☒	☐	Animals and other organisms
☒	☐	Human research participants
☒	☐	Clinical data
☒	☐	Dual use research of concern

Methods

n/a	Involved in the study	
☒	☐	ChIP-seq
☒	☐	Flow cytometry
☒	☐	MRI-based neuroimaging

Antibodies

Antibodies used	Western blotting for RNF4 used and in-house antibody raised in chicken as described (52). Immunofluorescence analysis used a monoclonal antibody to RNF4 (54) and an in-house antibody to PML raised in chicken (53).
Validation	RNF4 chicken antibody validated for Western blotting by lack of reactivity against extracts from RNF4-/- cells. RNF4 monoclonal antibody validated for immunofluorescence by lack of reactivity against RNF4-/- cells. PML chicken antibody validated for

immunofluorescence by lack of reactivity against PML-/- cells.

Eukaryotic cell lines

Policy information about cell lines

Cell line source(s)	U2OS RNF4-/- cells were derived in house using CRISPR/cas9 technology and were described previously (52)
Authentication	Genotyping as described (52)
Mycoplasma contamination	Cell lines were tested for mycoplasma contamination and found to be negative.
Commonly misidentified lines (See ICLAC register)	no commonly misidentified cell lines were used in this study.
