# Supplementary figures and images for: Functional 3D architecture in an intrinsically disordered E3 ligase domain facilitates ubiquitin transfer
Source: Nat Commun. 2020 Jul 30;11:3807. doi: 10.1038/s41467-020-17647-x (PMC7393505; doi:10.1038/s41467-020-17647-x)

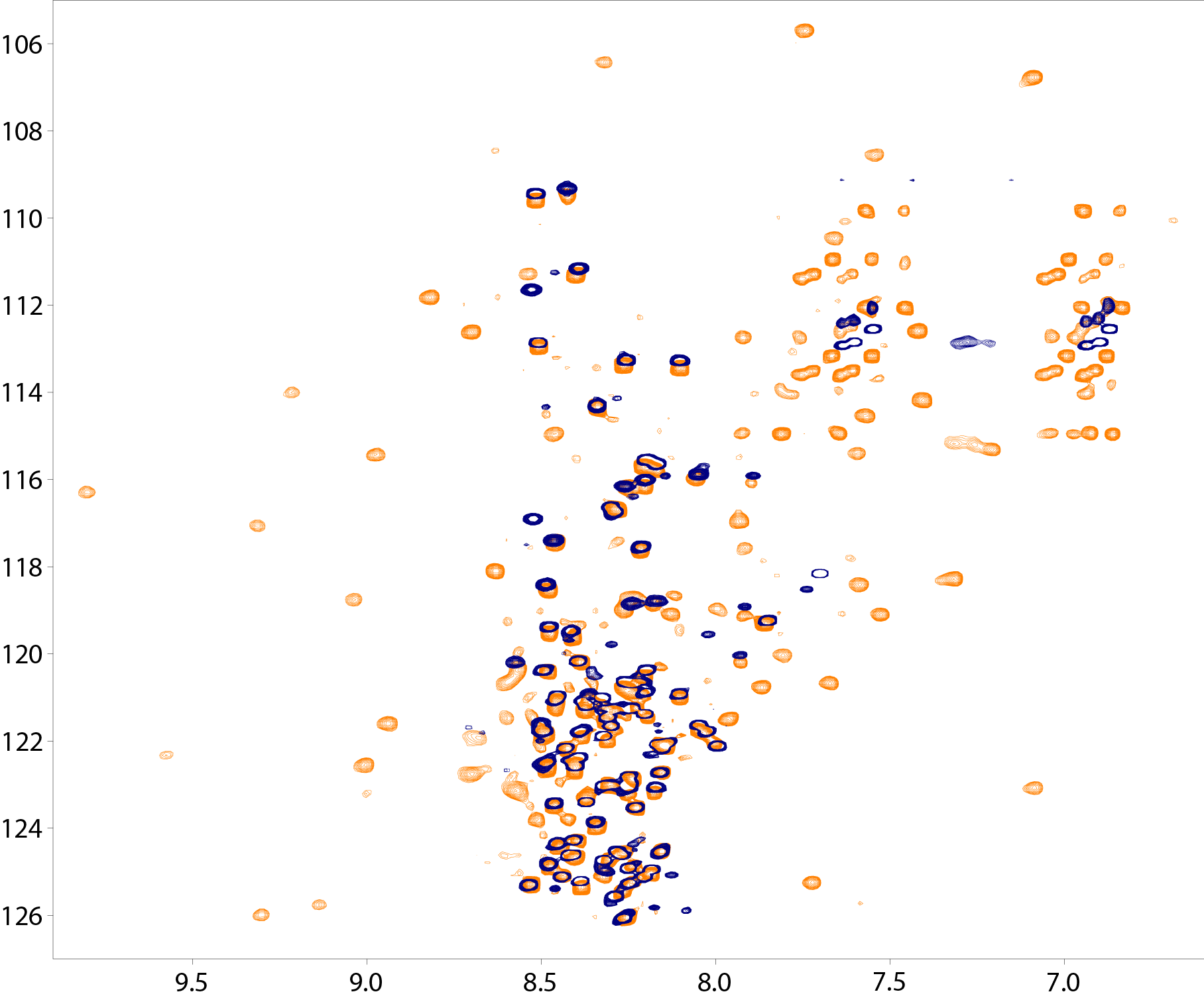

Supplement: Supplementary file 4 — Source Data [file 41467_2020_17647_MOESM4_ESM.zip › Source data/Fig. 1/overlayrnf4.png]

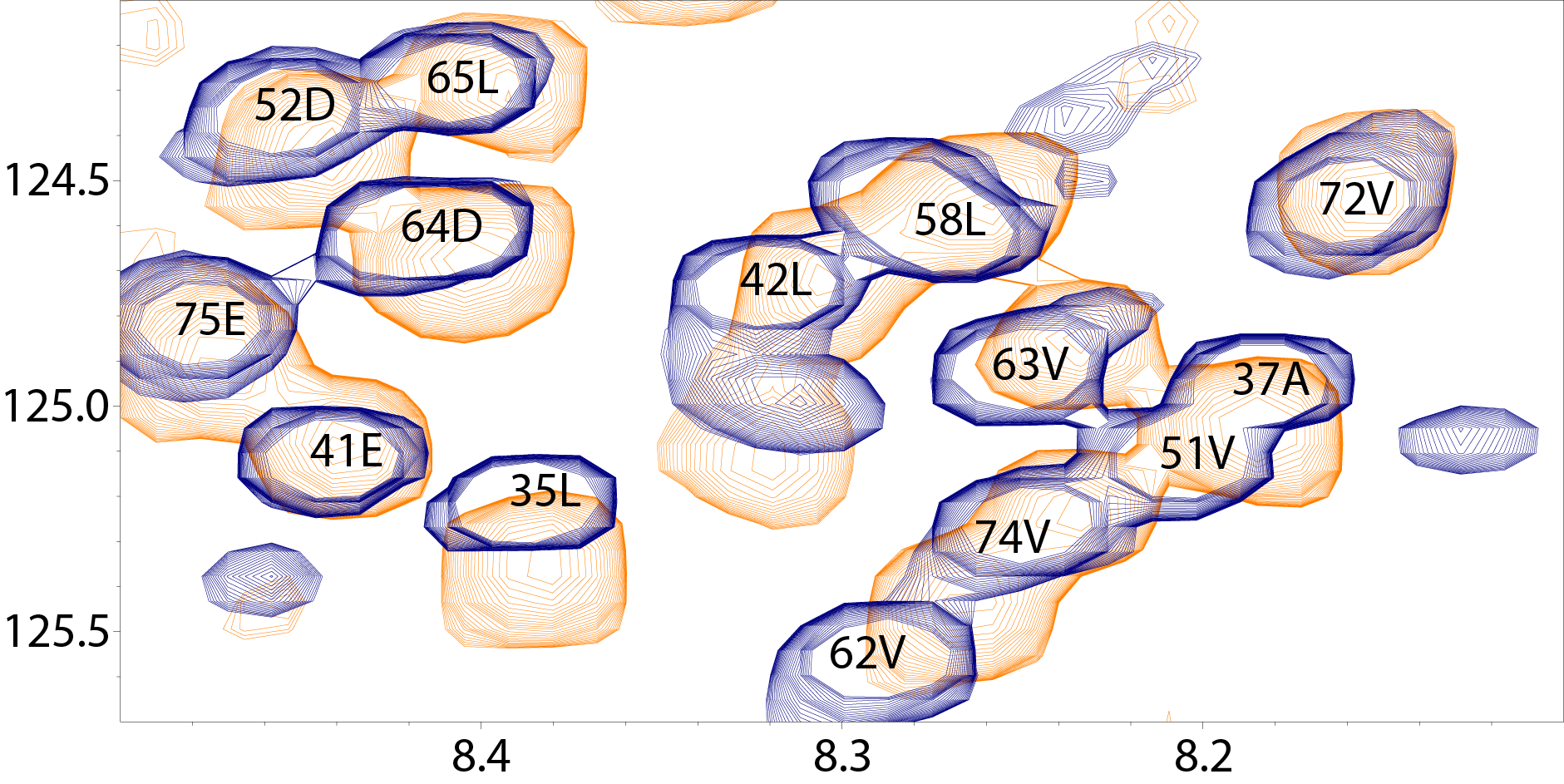

Supplement: Supplementary file 4 — Source Data [file 41467_2020_17647_MOESM4_ESM.zip › Source data/Fig. 1/overlayrnf4_blowout.png]

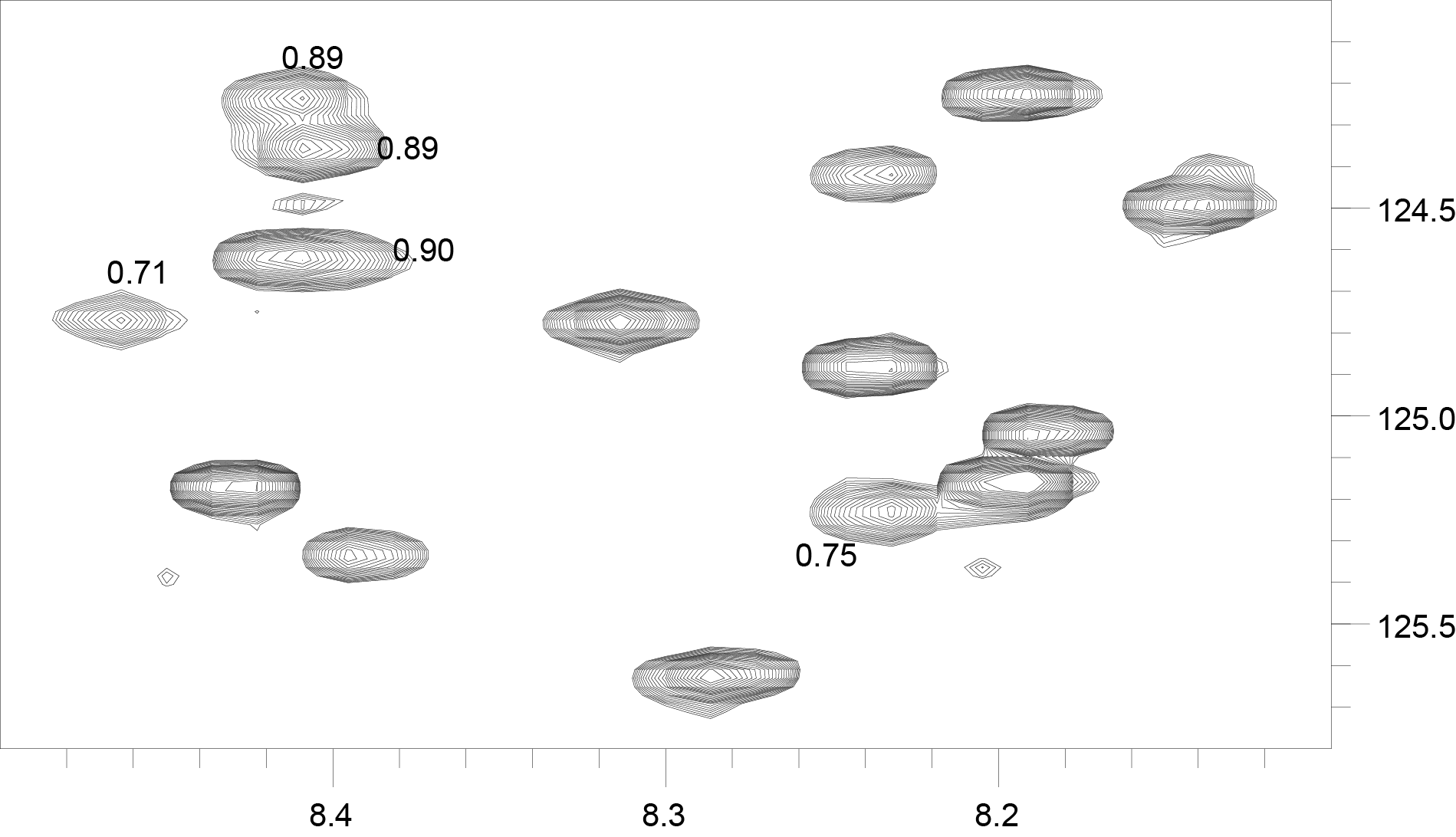

Supplement: Supplementary file 4 — Source Data [file 41467_2020_17647_MOESM4_ESM.zip › Source data/Fig. 4/Figure 4B/hsqc_mtsl_Cter.png]

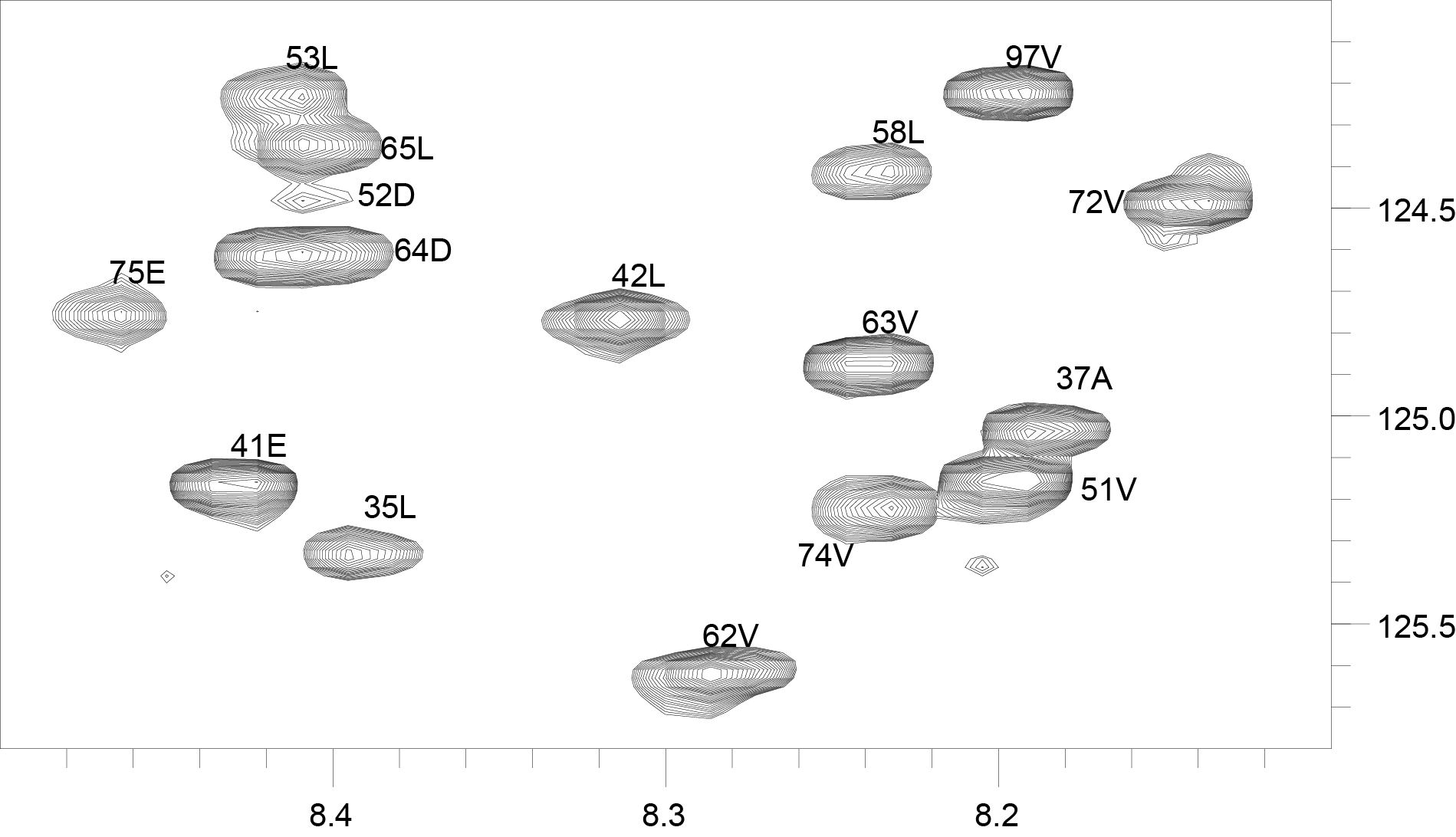

Supplement: Supplementary file 4 — Source Data [file 41467_2020_17647_MOESM4_ESM.zip › Source data/Fig. 4/Figure 4B/hsqc_reduced_Cter.png]
