# Supplementary material for: Functional 3D architecture in an intrinsically disordered E3 ligase domain facilitates ubiquitin transfer
Source: Nat Commun. 2020 Jul 30;11:3807. doi: 10.1038/s41467-020-17647-x (PMC7393505; doi:10.1038/s41467-020-17647-x)
Supplement: Supplementary file 4 — Source Data [file 41467_2020_17647_MOESM4_ESM.zip › Source data/Supp fig 11-15/allspectra_NMR.pdf]

C-terminal reduced

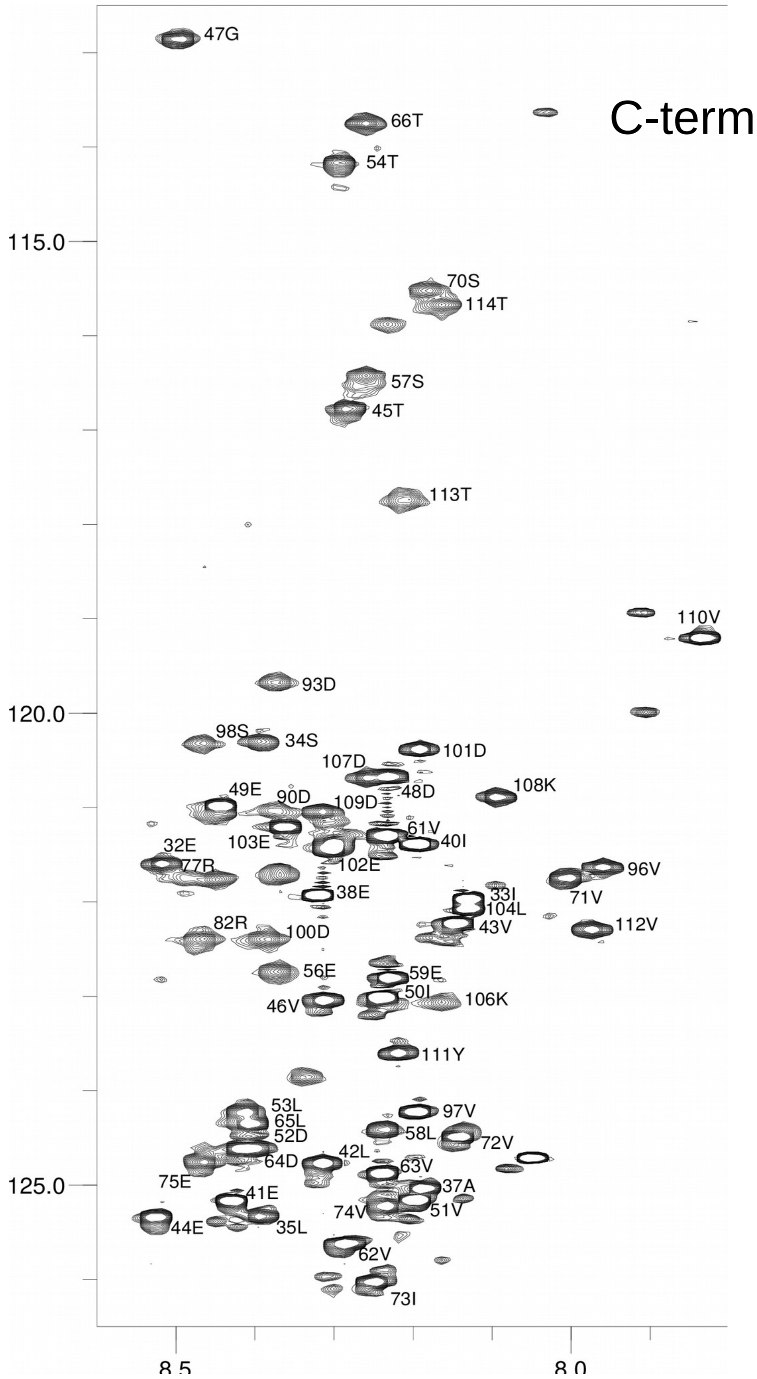

C-teminal spin-labelled

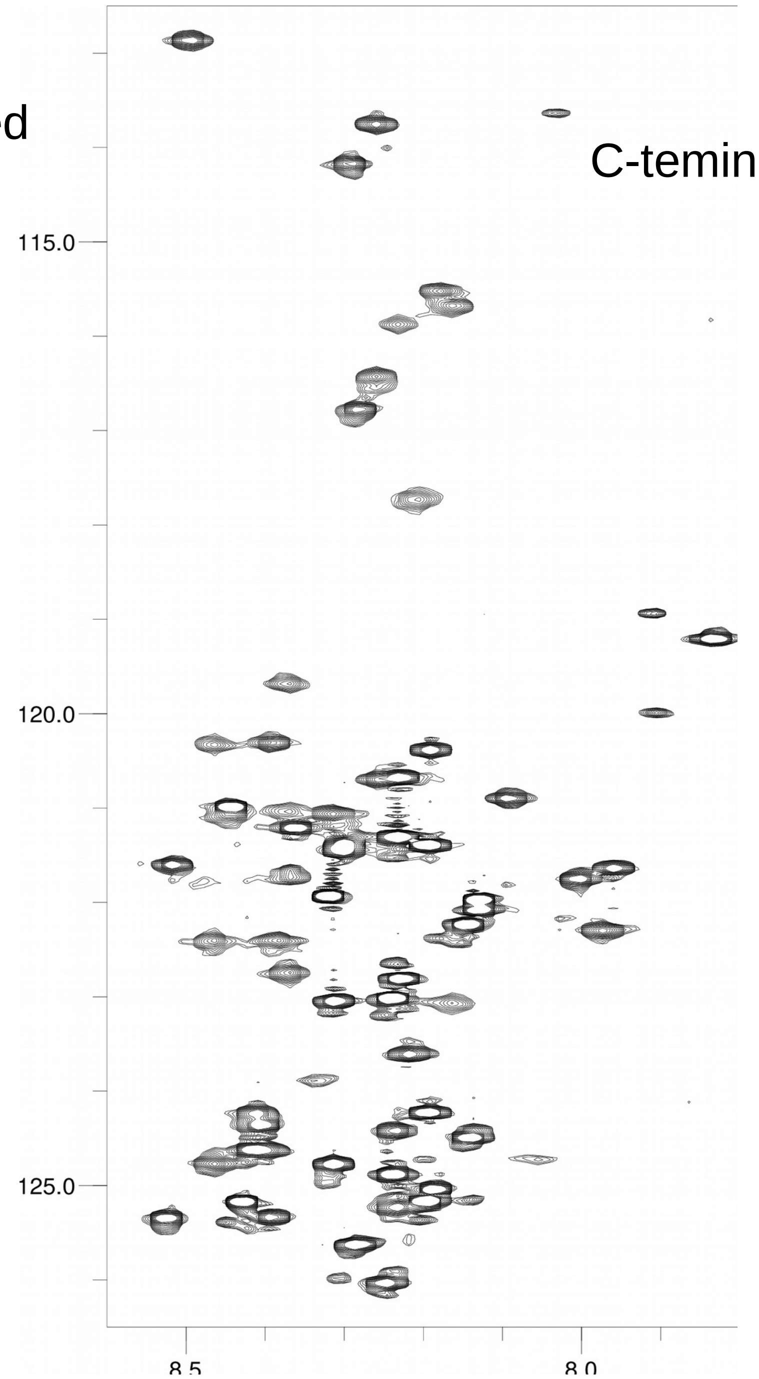

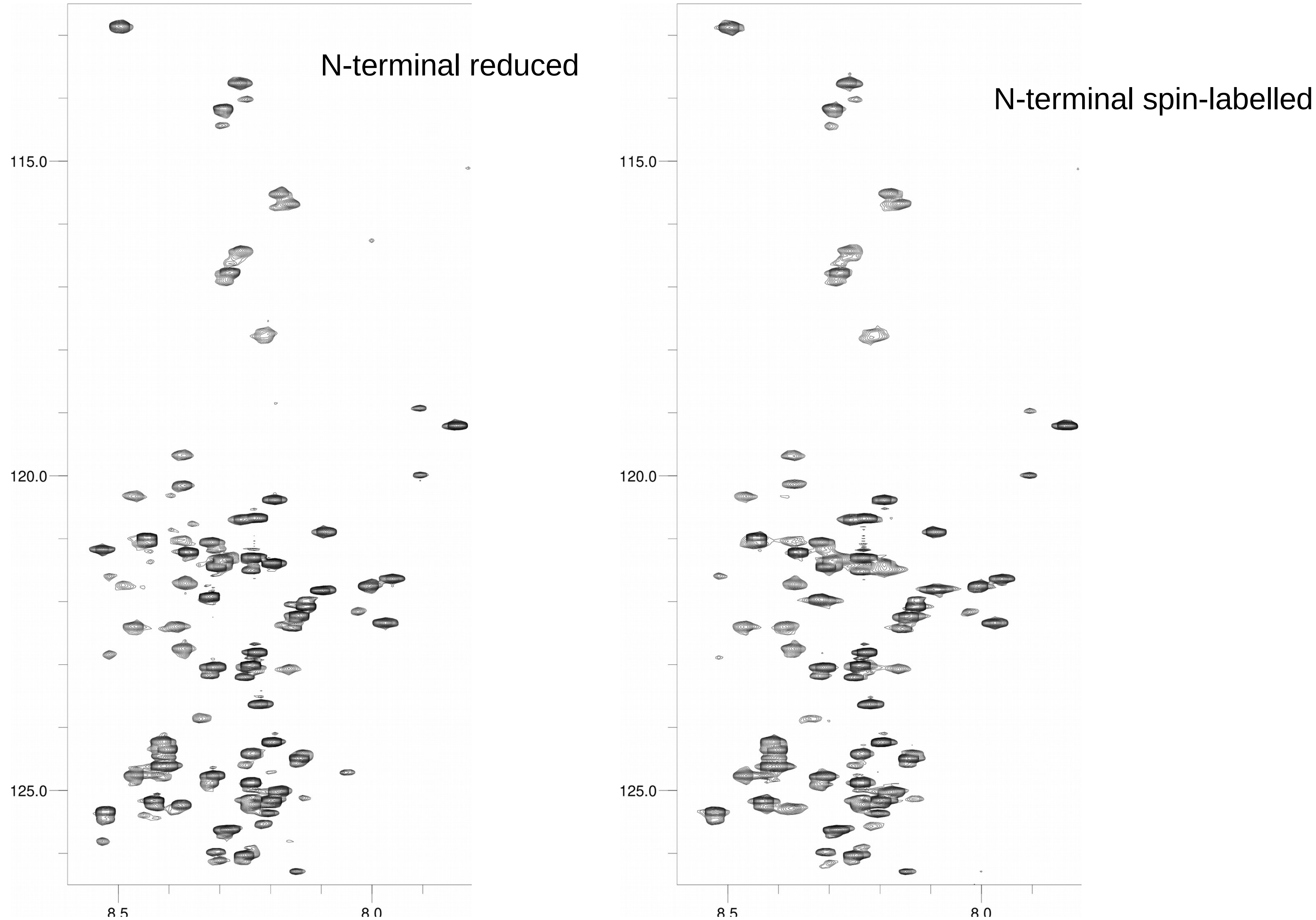
N-terminal reduced
N-terminal spin-labelled
115.0
120.0
125.0
8.5
8.0
115.0
120.0
125.0
8.5
8.0

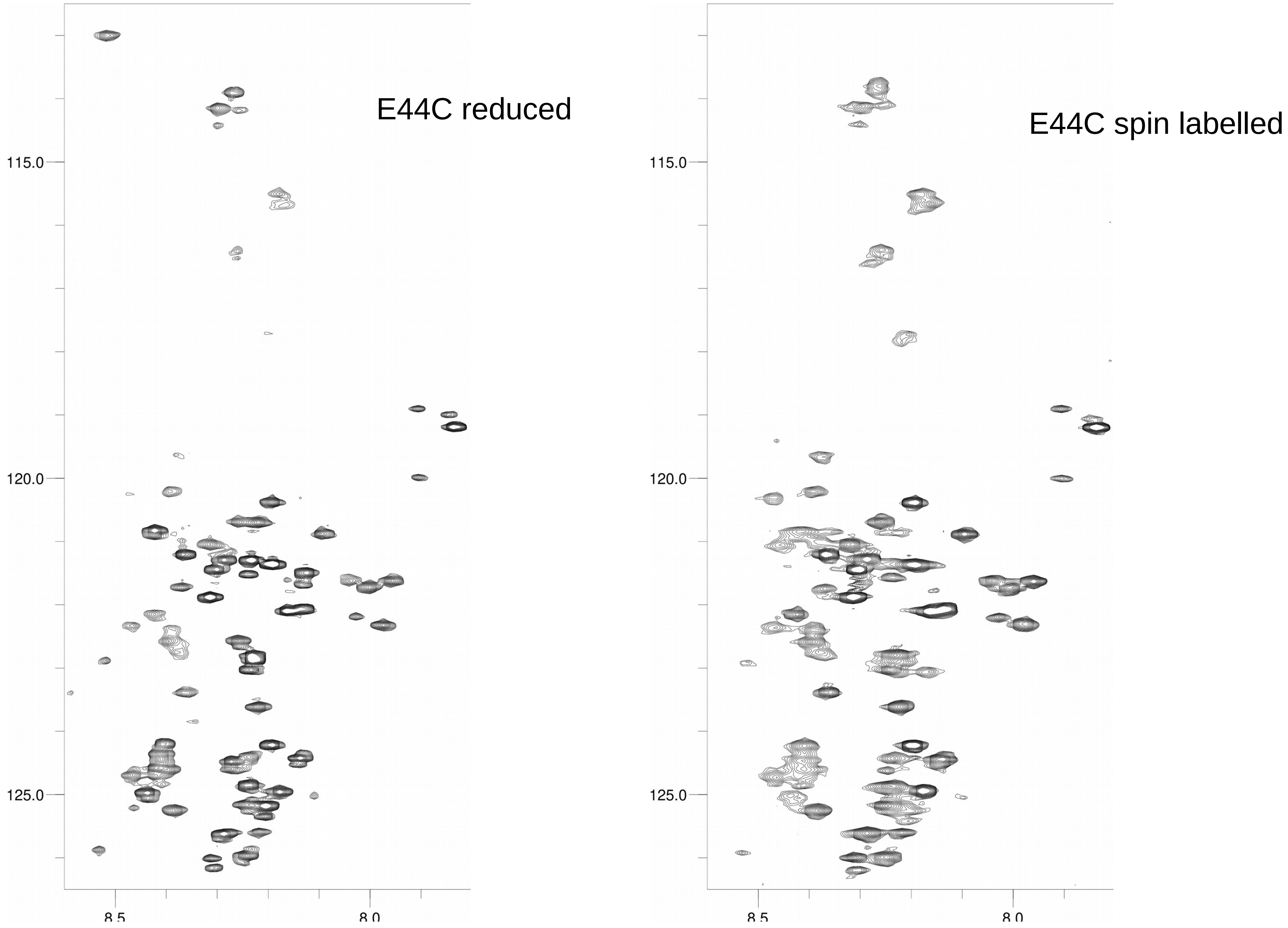
E44C reduced
E44C spin labelled
115.0
120.0
125.0
8.5
8.0
115.0
120.0
125.0
8.5
8.0

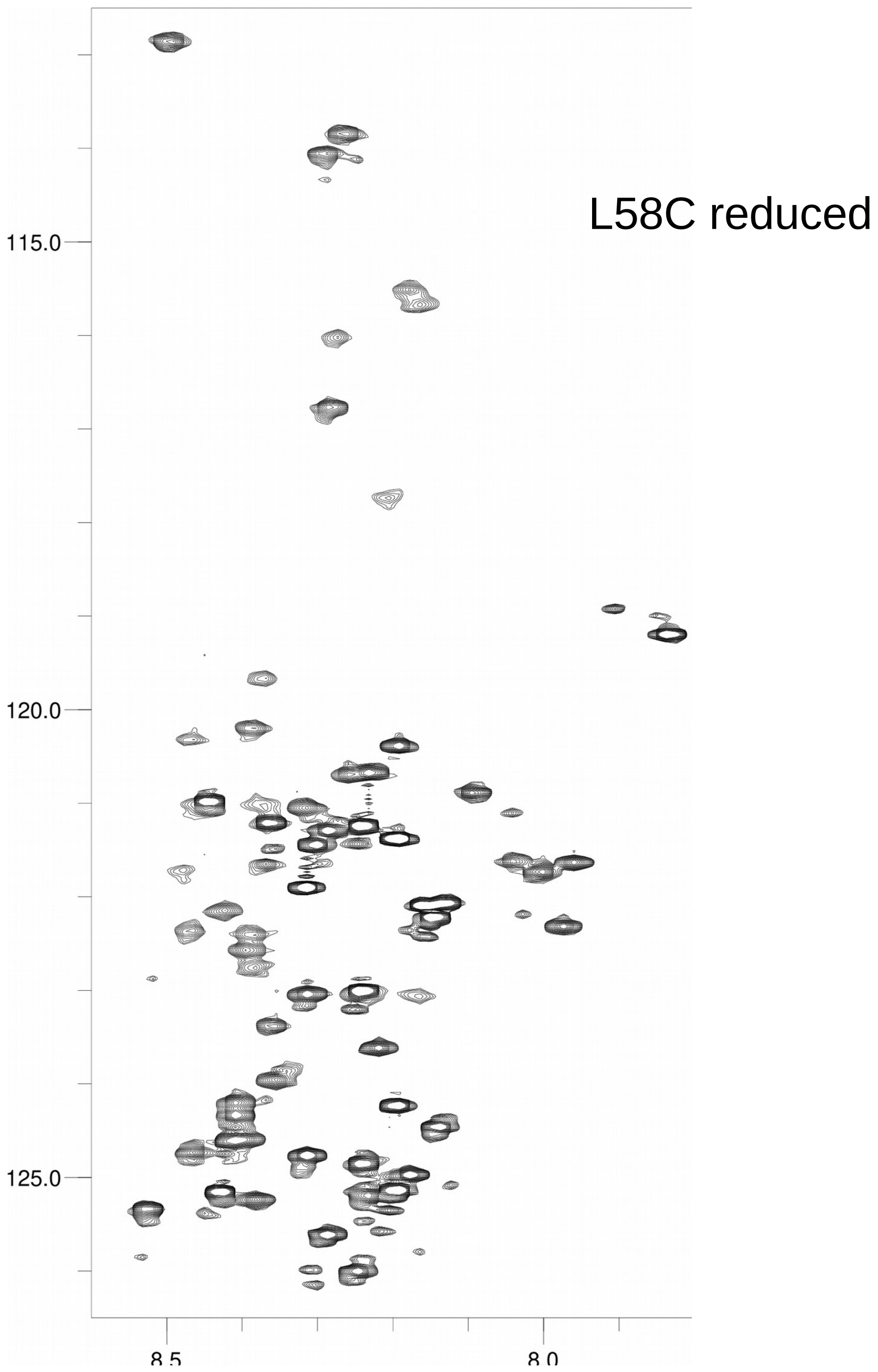
L58C reduced
115.0
120.0
125.0
8.5
8.0

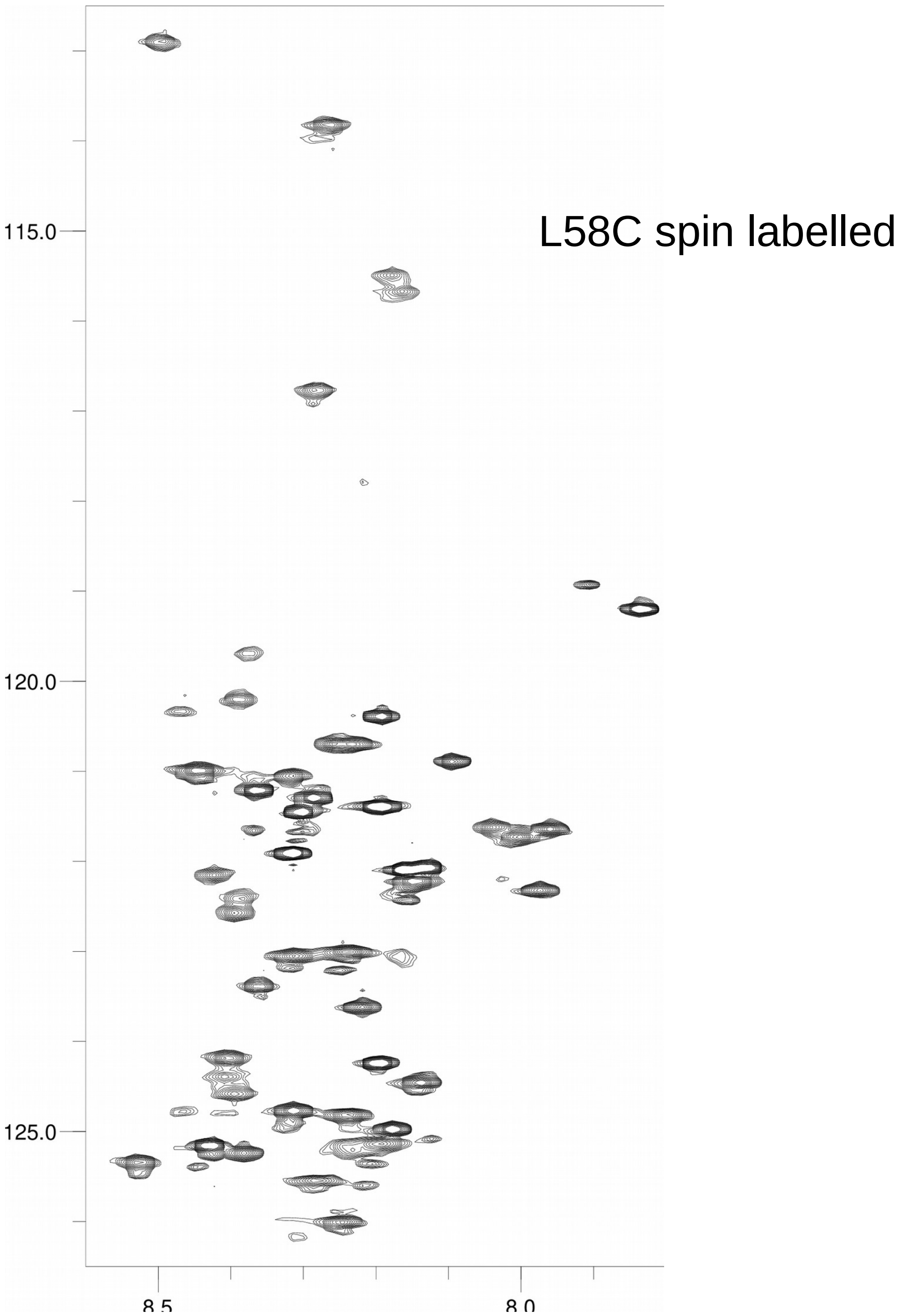
L58C spin labelled
115.0
120.0
125.0
8.5
8.0

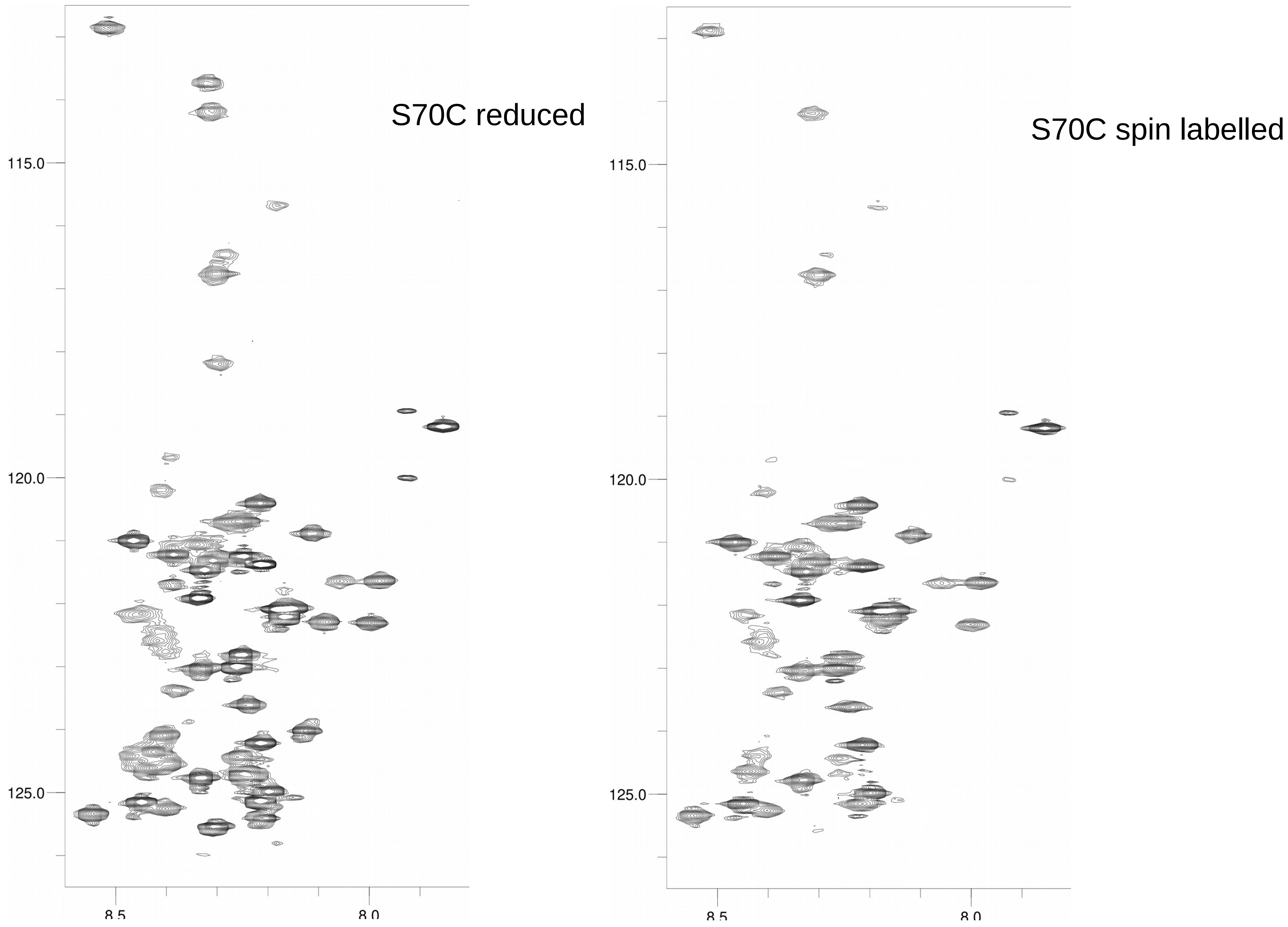

S70C reduced
S70C spin labelled
115.0
120.0
125.0
8.5
8.0
115.0
120.0
125.0
8.5
8.0
